# Gold Decoration To Improve Rh-Nanoalloys for CO-Adsorption

**DOI:** 10.1021/acs.jpcc.4c08091

**Published:** 2025-04-24

**Authors:** Letícia F. Basso, Mirko Vanzan, Laura Prati, Vagner A. Rigo, Francesca Baletto

**Affiliations:** † Department of Physics, 9304 University of Milan, 20133 Milan, Italy; ‡ Department of Chemistry, 9304 University of Milan, 20133 Milan, Italy; ¶ Department of Natural Sciences, 74354 Federal University of Technology - Paraná, Cornélio Procópio 86300-000, Brazil

## Abstract

After an accurate mapping of the available sites on Rh_19_ and three different Rh_19_Au_*M*_ nanoalloys (where *M* = 20, 36, or 52), the CO adsorption properties are obtained using density functional theory (DFT) at the PBE functional level, incorporating the Hubbard correction (PBE + *U*). The *U* values are selected to reproduce the correct adsorption trend and consistent binding of CO on Rh(111) in order to align with experimental measurements. The stabilization of the top adsorption site of CO, regarding the hollow one, is enhanced on the Rh_19_ surface, compared to the Rh(111) surface. Our findings indicate that the strength of CO adsorption on small AuRh nanoparticles can be effectively tuned by varying their morphology and composition. The energy landscape of the Rh-based nanoalloys is relatively flat, with the adsorption energies at all sites varying within 0.3 eV, similar to the trend observed for Rh(111), although Rh sites in the nanoalloys generally bind CO more strongly. Adding Au shortens the CO-Rh bond, and sites with a mixed chemical environment result in instability. The presence of Rh enables Au-sites to bind CO twice as strongly as in Au(111).

## Graphical abstract

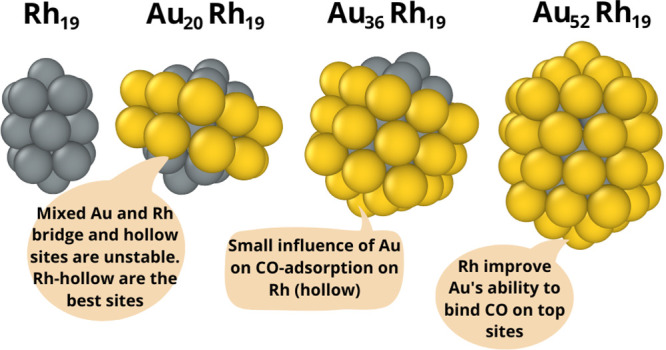

## Introduction

CO is a well-known harmful gas formed as a side product in many industrial processes that involve fossil fuel combustion.^1^ Finding a feasible way to convert CO into useful high-energy molecules, such as CH_4_ or short-chain alkanes, would likely reduce the chemical hazards of several industrial processes while producing useful and commercially relevant chemicals. In this direction, most modern chemical processes rely on reactions catalyzed by transition metals (TM). These catalysts typically provide high activity and recyclability but often suffer from poor selectivity and require significant energy to work efficiently.^2^ A promising way to overcome these limitations while maintaining or even improving the catalytic capabilities is by using TM nanoparticles (NPs) and nanoalloys (NAs) as catalysts.^3^ Metallic NPs and NAs exhibit pronounced quantum confinement effects due to their ultrasmall size (especially below 2 nm), resulting in a unique optoelectronic response, magnetism, and catalytic features.^4–11^ Furthermore, poorly miscible metals can alloy at the nanoscale, leading to systems with distinct structural and electronic properties,^12–14^ and studies on bimetallic and segregated surfaces show that the interaction energy can be tuned accordingly to the chemical composition.^15,16^

Rhodium nanostructures have recently attracted the attention of the nanoscience community as they may support the generation of hot carriers upon excitation of Localized Surface Plasmon Resonance (LSPR).^17^ Moreover, it has been demonstrated that Rh-NAs can promote complex reactions such as water splitting,^18^ H_2_O_2_ synthesis,^19^ NO reduction,^20,21^ and CO_2_ methanation.^22–25^ There is solid evidence that Rh surfaces and Rh-NPs can strongly bind pollutants such as NO and CO, making Rh an ideal candidate as a catalyst for their transformation and capture.^26–30^ Aligned with these results, by measuring the shift in CO-stretching frequency by infrared spectroscopy, an enhancement of the CO-Rh_*N*_ binding strength is observed up to *N* ∼ 19.^31,32^ This is explained by the CO-NP back-donation on low coordinated sites, and the same authors suggest that the weakening of the CO bond decreases at larger sizes.^31,32^ On Rh_*N*_ with *N* between 3 and 15, the top is the most favorable adsorption site, but bridge and hollow sites occur, mainly on Rh_12_ and Rh_13_.^33^ CO binding occurs on top, hollow, and bridge sites for NPs with 37 atoms^34^ and even at *N* = 201.^28^ Experiments combined with Density Functional Theory (DFT) calculations on Rh_55_ indicate a 3-fold hollow as the most stable site. However, the energy landscape shows that the top and bridge sites along the edges are very close in energy.^35^

From the pioneering work by Haruta,^36^ supported gold NPs and NAs have been extensively studied for CO oxidation.^37–39^ Besides, AuRh NAs are suggested as an efficient catalyst for many light-induced reactions.^40–42^ Freire et al. studied the adsorption of Rh on Au(111) surface, showing that Rh adatoms donate charge (∼0.13*e*) to gold atoms and that the presence of Rh can induce some strain of the gold surface layer due to their atomic radius mismatch.^43^ DFT calculations on AuRh NAs confirm that charge separation is due to the different electronegativity of the two metals.^44^ AuRh-NAs, within 1–3 nm, showed CO oxidation at low temperatures and good resistance to deactivation^45^ for CO transformations.^46^ Although AuRh tends to form segregated structures with an Au shell covering a Rh core,^47,48^ experimental measurements detect that the presence of CO could cause surface segregation of Rh atoms, which are more prone to bind the molecule.^49^ The strongest CO adsorption energy on the top sites is −2.15 and −1.12 eV on Rh_38_ and Au_38_, respectively.^40^ The same authors evaluated the adsorption of CO on alloyed AuRh-NPs. They showed that the adsorption energy is stronger when the CO molecule is on top of rhodium atoms if compared to gold, which is connected to a reduced charge transfer to CO while on Au.^40^

This work addresses the influence of different Au loadings on Rh. We provide an in-depth analysis of the interactions between CO and Rh(111), specifically focusing on a Rh_19_ double icosahedron and three different Au_*M*_Rh_19_ configurations, where *M* is equal to 20, 36, or 52. Based on our previous work on the growth of AuRh,^44^ we selected three stable configurations: an Au scarf decoration with Au_20_Rh_19_, a ball-cup structure with Au_36_Rh_19_, and a chiral Au shell consisting of 52 atoms over the Rh_19_ seed. Given the negligible miscibility of Au in Rh, we expect that Au will segregate in the AuRh nanoalloys. In the following, nanoparticle refers to Rh-only objects, while nanoalloys refer to AuRh nanostructures.^50^ We adopt a standard nomenclature to identify chemical ordering when surface segregation appears, as for Janus, ball-cup, and core@shell nanoalloys.^50,51^ We map the available sites on Rh and AuRh nanoalloys by the strained generalized coordination number.^52^ We estimate the adsorption energy using DFT-based calculations without and with the Hubbard correction.^53^ The addition of the Hubbard has been proven to correctly model CO interaction with Rh-based systems.^54^

We show that the CO molecule strongly adsorbs onto a Rh double-icosahedron with 19 atoms at various sites, with the atop site being the most favorable. However, the energy landscape seen by the CO is relatively flat. In all cases, we observe an elongation of the C–O bond length compared to that of the isolated CO molecule. Focusing on AuRh NAs, bridge and hollow sites formed by a mixture of Au and Rh atoms result in instability. The presence of Rh-atoms close to or underneath Au strengthens the CO-Au bonding on midcoordinated sites, peaking at −1.12 eV at the PBE level (∼ −1 eV at PBE + *U*, with *U* = 2.6 eV). The paper is organized as follows. We dedicate a section to presenting a summary of the CO-puzzle on TM surfaces, focusing on Rh(111). This section supports the choice for a PBE + *U* scheme, discussed in Methodology. Then, we show the CO adsorption results on Rh(111). Finally, we present the results of the CO adsorption on Rh NP and AuRh NAs.

### CO on Transition Metals: A DFT Puzzle

Tackling the CO-TM interaction has been proven to be challenging when the XC functional is either at the Local Density Approximation (LDA) or Generalized Gradient Approximation (GGA).^55–57^ Three strategies have been proposed to predict the correct order for CO-adsorption on TM surfaces: hybrid functionals, calculations at higher levels of theory (e.g., RPA), and Hubbard corrections.^58–62^

Regarding the adsorption on Rh(111), PBE calculations predict the hollow as the most stable adsorption site even in the low coverage limit.^56^ At the same time, experiments show CO adsorbed on top sites up to the coverage limit of 0.25 ML, with a desorption energy of 1.61 ± 0.05 eV.^63^ Other experiments agree with the top mode and adsorption energy between 1.3 to 1.7 eV.^64,65^ Hollow sites become the most favorable only above 0.75 ML according to Linke et al.^63^ and 0.33 ML according to Frank and Bĺaumer.^66^ The failure of DFT to reproduce CO adsorption occurs on many TM surfaces such as Pt(111),^60^ Cu(111),^61^ Cu(100),^67^ and Rh(100),^55^ leading to the well-known *“CO-puzzle on TM surfaces”*.^60,68,69^ There is a consensus about the reason behind the failure of GGA-DFT, which is due to the CO-TM bond nature that drives electrons from the CO 5σ molecular orbitals to the metal bands, followed by an electron back-donation from the metal to the CO 2π* orbital.^15,58,70–74^ Because of the intrinsic electronic self-interaction, DFT tends to increase the interaction between the CO 2π* and the *d*-band of the TM. Electron back-donation increases more for highly coordinated hollow sites than top ones.^57,58,60,61,75^ Some GGA functionals, such as RPBE,^55^ improve this feature. However, RPBE predicts the TM bulk and surface structural and electronic features worse than other GGA-functionals.^24,76^

Stroppa et al. showed that hybrid PBE0 and HSE03 predict the correct adsorption order on Cu(111) and Rh(111) but fail on Pt(111).^75^ However, the predicted adsorption energies still differ from the experimental values more than the standard PBE functional. Moreover, PBE better describes pristine Rh NPs compared to the hybrid HSE.^77^ B3LYP reproduces the experimental energy values of CO on the TM slab but underestimates the C–O bond distance, with the molecule losing charge upon adsorption.^70^ Random Phase Approximation (RPA) correctly predicts top adsorption on Rh(111) at low coverages.^56^ The same authors showed that the bridge site becomes the most stable for coverage of 0.5 ML and the hollow one above 0.75 ML.^56^ The CO–CO interaction on the surface is claimed to be the main reason behind modifications in the relative stability of the adsorption sites. Despite its accuracy, the RPA scheme is computationally costly. To the best of our knowledge, there is no evidence that this methodology would work on metallic and bimetallic NPs. Similar considerations are made with higher-level computational approaches, such as coupled-cluster (CC) and configuration-interaction (CI) methods. Mason et al.^67^ used CC and CI methods to relocate the molecular energy levels of CO, providing the top as the most stable site on Pt(111), Rh(111), and Cu(111). However, their application to metallic NPs is prohibitive, and they have not been considered further.

We emphasize that the CO-puzzle pertains to the estimation of adsorption energy, while system geometry and vibrational frequencies are well reproduced by PBE.^59–61,63^ Interestingly, this remains true when Hubbard-U corrections are included in the PBE Hamiltonian. It has been demonstrated that adding the Hubbard correction to PBE using the standard scheme from Dudarev^78,79^ (PBE + *U*) can resolve the CO-puzzle effect.^24^

Dabo et al. calculated that bond length and stretching frequencies of CO in the gas phase are within ∼1% of experiments.^60^ Furthermore, PBE + *U* gives the geometrical properties of CO adsorbed on Pt(111), Cu(111), and Rh(111) in excellent agreement with measurements.^75^ Patra et al.^57^ predict the correct energetics for CO on Pt(111) using simply the *U*-corrected charge density (see Supporting Information). On Rh(100), Vanzan et al.^24^ applied PBE + *U* to study CO adsorption and dynamics, suggesting the bridge site as the most stable.

### Methodology

We choose the Rh_19_ and three stable Au-decorations with an Au loading between 50 and 75%: the “scarf”, Au_20_Rh_19_; a ball-cup with 36 Au atoms; and a complete Au-shell at Au_52_Rh_19_. Our choice stems from their remarkable stability and likelihood of being synthesized from coalescence, one-by-one growth, and annealing in inert-gas cluster sources.^44,80^ On Rh(111), we compare four known adsorption modes: top, bridge, hollow 3-fold or hcp, and 4-fold or fcc. After a visual identification, we map nonequivalent sites on Rh_19_ and Au_*M*_Rh_19_, with *M* = 20, 36, 52, using the generalized coordination number (GCN) and the strained generalized coordination number (sGCN) of a surface site.^52,81^ GCN­(α) considers the coordination of the second nearest neighbors of a surface site to describe the coordination of a given surface site, showing improved results against simple coordination for nanomaterials.^52^ Similarly, sGCN accounts for the strain effects on GCN, due to modifications in the bond distances with regard to the bulk crystal. For a detailed discussion and equations, see the Supporting Information. For these calculations, we employ our homemade characterization software.^82^ We stress that an accurate mapping of NP’s sites is needed to extract a structure–property relationship (see Figure S8 in the Supporting Information).

The adsorption energy reads

$$E_{\mathrm{ad}}=E_{\mathrm{sys}+\mathrm{CO}}-E_{\mathrm{sys}}-E_{\mathrm{CO}}$$ 1

where *E*_sys+CO_ is the total energy of the molecule adsorbed on the metallic systems, *E*_sys_ is the total energy of the Rh surface, Rh_19_, or Au_*M*_Rh_19_. *E*_CO_ is the total energy of the isolated CO molecule in a vacuum.

To calculate the total energy, we employ ab initio calculations, as implemented in the Quantum-ESPRESSO package, version 7.1,^83,84^ using PBE as reference XC functional.^85^ We estimate the adsorption energy of fully relaxed geometries at the PBE level, including the Hubbard correction (PBE + *U*) to the CO molecule. We detail our choice in the next section and in the Supporting Information.

We employ norm-conserving pseudopotentials^86^ to describe the interaction of ionic cores with valence electrons, where the electronic configuration of isolated Au, Rh, C, and O atoms is 6*s* ^1^5*p* ^6^5*d* ^10^, 5*s* ^1^4*p* ^6^4*d* ^8^, 2*s* ^2^2*p* ^2^, and 2*s* ^2^2*p* ^4^, respectively. An energy cutoff of 60 Ry is applied, whereas 400 Ry is the cutoff for the charge density. According to convergency tests, the adsorption energy of CO on Rh(111) changes by less than 0.9 meV if the cutoff is 90 and 630 Ry, respectively. In all cases, a Gaussian smearing of ∼0.01 eV is imposed on electronic occupation.^87^ A vacuum region of 20 and 15 Å is added for slabs and nanostructures, respectively. A cubic box with a length of 30 Å is used to calculate the energy of the free CO molecule. For isolated systems in a cubic box, long-range Coulomb interactions between periodic images are treated using the Makov-Payne correction.^88^

By optimizing the energy of an fcc unit cell containing a single Rh atom using an 8 × 8 × 8 *k*-point mesh, we retrieve an Rh-bulk lattice parameter of 3.83 Å, in agreement with previous PBE calculations^76^ and less than 1% greater than the experimental value.^89^

Rh­(111) slabs are carved from bulk calculations, considering a 2 × 2 replica from the minimal unit cell along the *x* and *y* periodic directions and 4 layers (2 × 2 × 4), corresponding to 6.53 Å along the *z*-direction. In the Supporting Information, we provide details for a (2 × 2 × 6) slab, corresponding to a 10.95 Å thickness. We calculate an energy difference of ∼13 meV for CO adsorbed top on a 2 × 2 and a 3 × 3 Rh(111) slab, in agreement with Liu et al.^56^ Being a marginal difference, we opt for a 2 × 2 to save computational time.

Rh-based nanoalloys are fully optimized with a Broyden–Fletcher–Goldfarb–Shanno algorithm with a force convergence threshold of 10^–5^ Ry/Bohr, and an energy convergence threshold of 10^–5^ Ry. We performed calculations at the Γ-point for the isolated systems, and we used a 7 × 7 × 1 *k*-points grid for the Rh(111) surfaces.

Extended Rh(111) slabs are not magnetic, but Rh-based NAs display a nonzero magnetization.^44,90^ In the case of Rh_19_ and Au_*M*_Rh_19_, we run spin-polarized calculations where each metal atom possesses a starting magnetization of 0.5 μ_B_ in a ferromagnetic order, being allowed to optimize along the calculation.

We calculate a few key quantities to check the feasibility of the considered Rh systems in binding CO. First, the charge redistribution per each atom *i*, Δ*q*_*i*_. This is the difference of the atomic Bader charge after (*q*_after_^*i*^) and before (*q*_before_^*i*^) adsorption:

$$\Delta q^{i}=q_{\mathrm{after}}^{i}-q_{\mathrm{before}}^{i}$$ 2

Bader charges are calculated using the code developed by the Henkelman Research Group, University of Texas at Austin (Texas, USA).^91,92^ For all systems, we calculate the changes of the CO bond length, Δ*d*_CO_, and the average bond length, *d*_M–C_, that C forms with the metal atoms. On Rh(111), we estimate the local rearrangements of Rh induced by CO, Δ*d*_Rh–Rh_, which is the average distance of Rh nearest neighbors in the uppermost layer. We label Δ*z* the induced surface roughness, calculated as the average Rh displacement perpendicular to the surface.

$$\Delta z=\sum_{i}\frac{z_{\mathrm{after}}^{i}-z_{\mathrm{before}}^{i}}{N_{\mathrm{surf}}}$$ 3

where *i* is the number of surface Rh atoms, and *N*_surf_ is their total number, e.g.,*N*_surf_ = 4 for the 2 × 2 slab.

## Results and Discussion

For the sake of clarity, we split our results into two subsections. One is dedicated to CO bound to a Rh(111) and the second to the chemisorption onto Rh-based NAs.

### CO on Rh(111)

Figure 1 shows the sites where CO can adsorb on an Rh(111). Table 1 lists the *E*_ad_ values and the relevant geometrical descriptors. At the PBE level, the hcp-hollow is the preferential site for CO-adsorption onto (2 × 2 × 4) Rh(111). The calculated adsorption energies align with previous calculations using similar computational setups. The wrong prediction of hcp-hollow as the most energetically favorable adsorption site agrees with the DFT results in the literature.^26,56^

**Figure 1:**
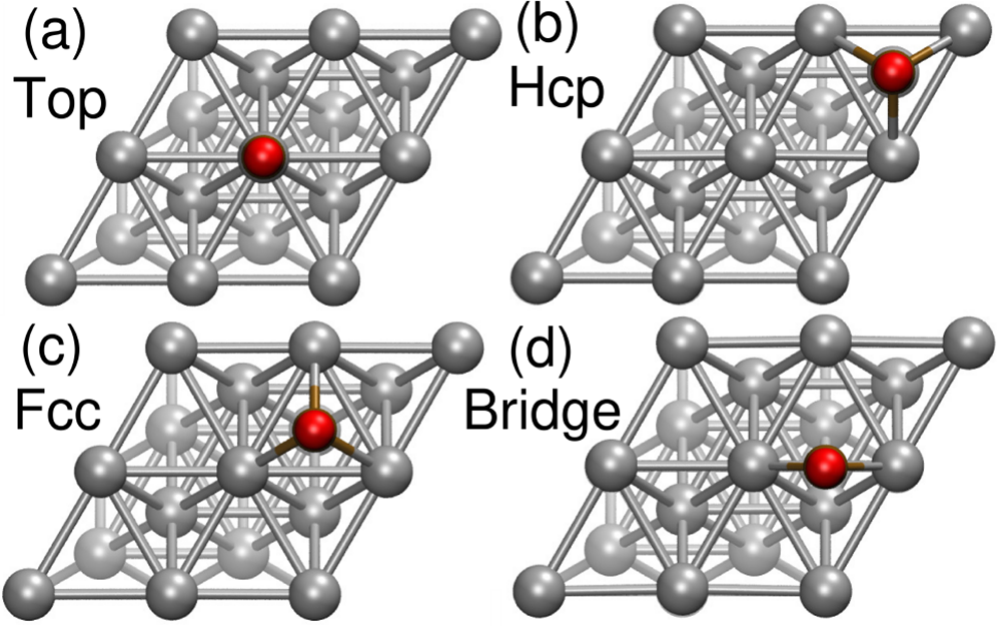
Pictorial representation of four adsorption sites (red balls) available on the Rh(111) surface: (a) top, (b) hcp-hollow, (c) fcc-hollow, and (d) bridge. The GCN characterization marks top with 7.5, bridge 6.67, 6.95, and 7.5 for fcc and hcp-hollow sites, respectively.

**Table 1:** Adsorption Energy (*E*_ad_) and Structural Descriptors for CO-(2 × 2 × 4) Rh(111)^a^

site	*E*_ad_	Δ*d*_C–O_	*d*_Rh–C_	Δ*d*_Rh–Rh_	Δ*z*
@PBE					
Top	–1.90	0.02	1.83	–0.001	0.047
Hcp	**-1.91**	0.05	2.09	0.028	0.033
Fcc	–1.83	0.05	2.11	0.013	0.042
bridge	–1.81	0.04	2.02	0.007	0.046
@PBE + *U*_1_					
Top	**-1.86**	0.02	1.84	–0.001	0.047
Hcp	–1.85	0.05	2.09	0.028	0.033
Fcc	–1.77	0.05	2.11	0.013	0.042
bridge	–1.76	0.04	2.02	0.007	0.046
@PBE + *U*_2_					
top	**-1.70**	0.02	1.84	–0.001	0.047
Hcp	–1.66	0.05	2.09	0.028	0.033
bridge	–1.57	0.04	2.02	0.007	0.046

a Δ*d*_C–O_ indicates the difference between the C–O bond length in the gas phase and when adsorbed (Δ*d*_C–O_ = *d*_C–O_^g^ – *d*_C–O_^ads^). *d*_Rh–C_ is the average Rh–C bond length, where the number of averaged bonds is site-dependent (1 for top, 2 for bridge, 3 for hollow sites). Δ*d*_Rh–Rh_ is the difference between the average Rh–Rh nearest-neighbor distances within the uppermost layer after and before adsorption. Δ*z* is the average difference in the height of the Rh outermost atoms after and before adsorption. *E*_ad_ is given in eV and distances in Å. We highlight the most stable site in bold. *U*_1_ refers to *U*-correction = 0.65 eV and *U*_2_ is for a correction of 2.6 eV.

The Hubbard scheme smoothly adjusts the *E*_ad_ to their experimental values.^63–65^ Figure S2, in the Supporting Information, shows the linear dependence of *E*_ad_ of CO on Rh(111) on the Hubbard corrections. However, the coefficient depends on the adsorption mode, and we observe a crossing between hollow and top modes. The experimental adsorbate stability ordering, with the top site being the most energetically favored even at 25% coverage, is reproduced by a *U*-correction as small as 0.65 eV (*U*_1_) on the 2*p* orbitals of C and O (see Table 1), in agreement with the *U*-value suggested by Köhler and Kresse.^54^ Nonetheless, a 2.6 eV *U*-correction (*U*_2_) is needed to reproduce the adsorption energy values within the experimental range. In addition, the energy difference between the top and hcp-hollow site enlarges to 40 meV with *U* = 2.6 eV, compared to only 10 meV using *U* = 0.65 eV (details in Supporting Information Figure S9). The PBE + *U* affects negligibly the geometry of the metal surface with respect to PBE. It is encouraging as PBE predicts geometry and CO-frequency stretching in agreement with experimental data.^60^ The top adsorption slightly enlarges the *d*_Rh–C_ bond length using PBE+U, compared with standard PBE, suggesting a reduction of the CO-surface interaction.

Figure 2 shows the DOS and pDOS of CO adsorbed on Rh(111). The complete pDOS for the isolated CO molecule is available as Figure S1 in the Supporting Information, whereas Figure S3 reports the pDOS of the pristine Rh(111) and Figure S4 reports the complete pDOS spectra for the CO top and hollow-hcp adsorbed on Rh(111). From the results, a CO 1π-Rh 4*d* orbital mix and a 2π*-Rh 4*d* interaction are visible, in agreement with other predictions.^93^

**Figure 2:**
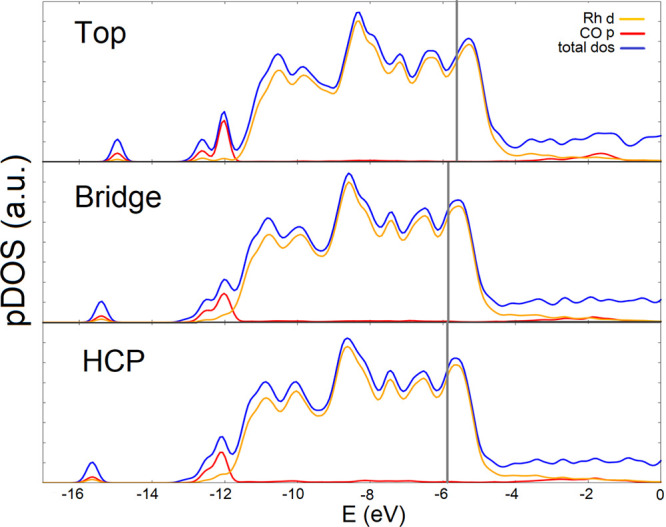
DOS and pDOS of Rh d and CO p orbitals of a Rh(111) slab with CO adsorbed on the top (upper panel), bridge (middle), and hollow-hcp (bottom) at the PBE + *U*_2_ level. Vertical lines refer to the position of the Fermi level for the three systems. We have aligned the DOS and pDOS to the vacuum level.

Although the three panels of Figure 2 look very similar, there are some differences depending on the adsorption mode: (i) The peak around −15 eV is very sensitive to the adsorption mode. We confirm that this peak is connected to the CO 4σ orbital, whose energy is strongly sensitive to the binding site. (ii) At −12 eV, a double peak stands for top adsorption. On the other hand, hollow-hcp has a higher peak at −10 eV. (iii) Although Rh 4*d* electrons are poorly affected by CO, the shoulder around −8 eV (Rh 4*d* and CO 5σ interaction) is more visible on the top than on the others. Figure S5 shows that different values of U do not sensibly change the DOS.

Because the relative displacements of Rh belonging to the subsurface layer are not significantly affected by CO (see Supporting Information, Table S1), the strain induced by adsorption is localized in the uppermost layer of the slab and in the molecule itself. The local effect is why a slab with a few layers provides a reasonable trend. After adsorption, the C–O bond length *d*_CO_^*^ is longer than the gas-phase length, *d*_CO_^g^ = 1.31 Å. It is 1.7 and 4.5% longer for the top and hcp-hollow sites, respectively. A longer elongation of the C–O bond for the hollow site compared to the top site is consistent with a stronger π backdonation at hollow sites^58^ (see Supporting Information, Figure S6). The average Rh–C bond distance, *d*_RhC_, is shorter for the top site than that for the hollow site by approximately 14%. This trend indicates a weakening of the C–O bond as the carbon atom increases its bond strength with the metal surface, i.e., Rh–CO. CO adsorption causes local distortions of the Rh(111) geometry, as seen in Supporting Information, Figure S7.

We estimate a displacement map after CO adsorption, with σ_*i*_ being the module of the atomic displacement vector σ_*i*_ = |*r⃗*_*i*_^after^ – *r̃*_*i*_^before^|. A nonzero σ_*i*_ per each atom *i* confirms that CO locally rearranges surface atoms. We note that the strain contributes to the adsorption energy, on the order of 0.06–0.13 eV for fcc hollow to top and 0.09 eV for bridge and hcp-hollow (see Supporting Information for a complete discussion). Because CO induces local distortions, the strain follows the surface roughness Δ*z* and the changes of the Rh–Rh distances underneath the site Δ*d*_Rh–Rh_; see Table 1. Essentially, Rh atoms that anchor the CO are lifted. For top adsorption, the amount of Rh-lifting depends slightly on the number of slab layers. The roughness sensibly changes in the 6-layer slab compared to the 4-layer one (see Supporting Information).

### Rh_19_-Based Nanoalloys

We consider four Rh_19_-based NAs,^44^ whose graphical representation is depicted in Figure 3. Rh_19_ is a double icosahedron (dIh)^94^ formed by four atoms lying on a 5-fold axis surrounded by three pentagons in such a way that the middle one is twisted for the other two as an hcp-hollow (or ABA) packing. A dIh is delimited by (111) facets only. The total magnetization of certain Rh_19_–CO configurations depends on the *U*-value, with a maximum difference of 2 μ_B_ from PBE to PBE+U2 (see the Supporting Information for more details). The Au_20_ scarf decoration of Rh_19_ induces, at first, a reconstruction of the inner Rh-seed. One of the rings of Rh_19_ rotates to form a decahedron compenetrated with an icosahedron (Dh_13_ + Ih_13_). The 20 atoms of Au form a “scarf” that covers the central ring, resulting in a (100) Au/Rh step on one side (in Figure 3, the A-view is taken from the 5-fold vertex of the Rh-Ih_13_) and a (111) Au/Rh step (in Figure 3, the B-view looks at the 5-fold vertex of the Rh-Dh_13_). Au_36_Rh_19_ has a ball-cup morphology,^40^ leaving just six atoms of Rh at the surface, including the 5-fold vertex of the Dh_13_ (B-view of Figure 3). In this structure, the inner Rh core is Dh_13_ + Ih_13_, and Au_52_Rh_19_ is a perfect Au-shell over Rh-dIh_19_, where the Au-shell is chiral. The bottom panel of Figure 3 shows the sites with a different sGCN or at least a different local chemical environment.

**Figure 3:**
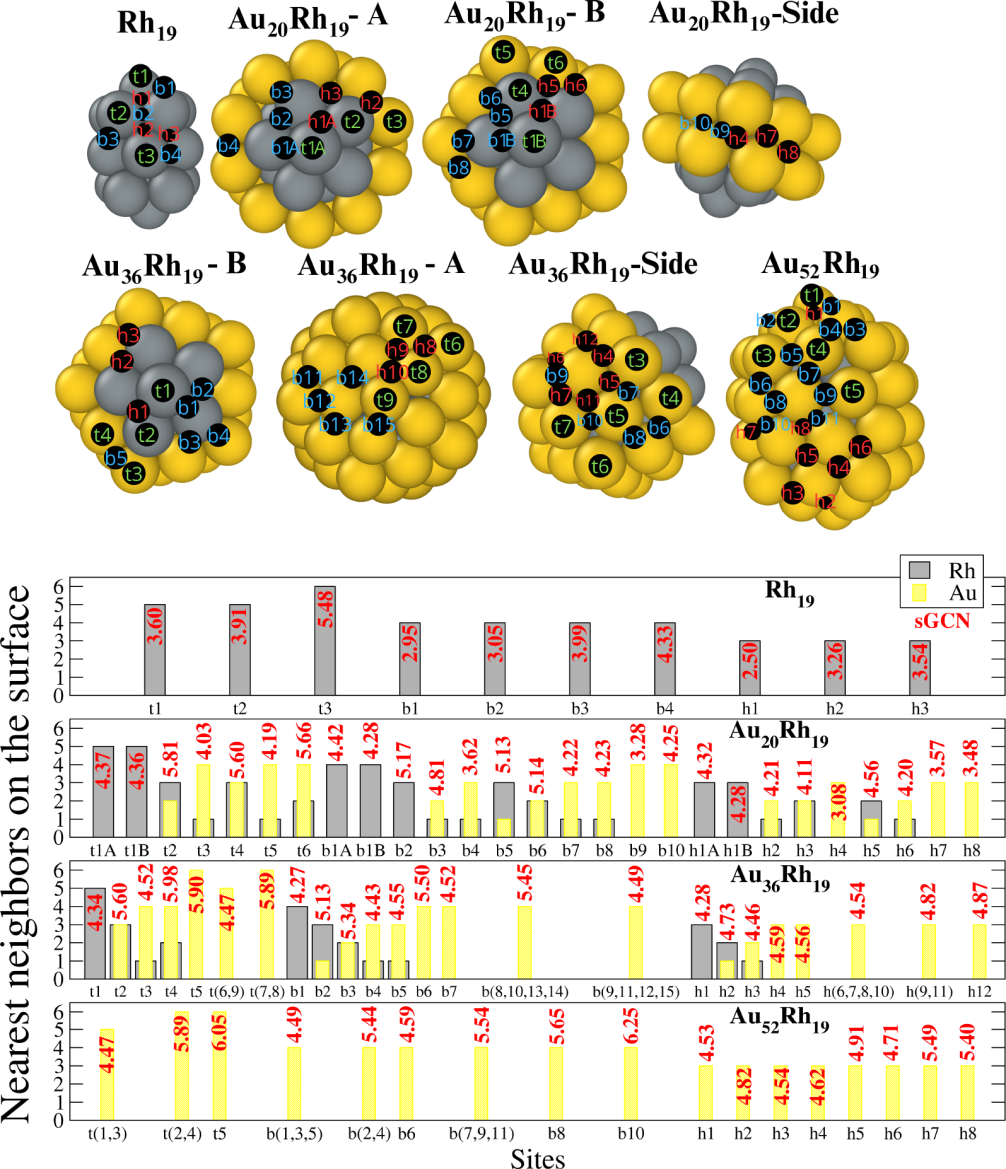
Upper panel shows the adsorption sites on Rh_19_, Au_20_Rh_19_ (A, B, and side view), Au_36_Rh_19_ (B, A, and side view), and Au_52_Rh_19_. Gold atoms are in yellow, and Rh is in gray. Sites are labeled “t” for top, “b” for bridge, and “h” for hollow, followed by an integer to list them. The bottom panel shows the local chemical environment of each adsorption site, with a gray (yellow) histogram counting the number of surfaces Rh­(Au) atoms surrounding the site. The sGCN values are in red.

Tables 2 and 3 list the adsorption energy for the various sites depicted in Figure 3 calculated at PBE, PBE + *U*_1_, and PBE + *U*_2_ levels. The value of the sGCN associated with the anchoring site is also included in the Tables. Some of the sites are unstable during PBE relaxation. These are highlighted by an → in Tables 2 and 3, with CO spontaneously moving to the indicated site. The Hubbard corrections do not qualitatively change our adsorption energy prediction. However, *U*_1_ = 0.65 eV reduces the strength of the adsorption by about 20–60 meV, while *U*_2_ = 2.6 eV lowers the adsorption energy by around 200 meV. As noted, the energy difference between the most stable top and hollow adsorption sites on Rh_19_ is 70 meV at PBE and PBE + *U*_1_, and 100 meV at PBE + *U*_2_. Therefore, the CO atop energetic preference is enhanced when adsorbed on Rh-NPs, compared to the extended Rh(111) surface. We estimate a U-dependency for the adsorption energy on t1 on Rh_19_ as *E*_ad_^PBE + U^ *@*t1 = *E*_ad_^PBE^ + 0.04*U*, while the slope for other sites is 0.06 ± 0.01 (see Supporting Information).

**Table 2:** GCN and sGCN of Adsorption Sites, and Adsorption Energy, *E*_ad_ (eV), of a CO Molecule on Rh_19_ and Au_20_Rh_19_ at the PBE, PBE + *U*_1_, and PBE + *U*_2_ Level of Calculation^a^

site	GCN	sGCN	PBE	PBE + *U*_1_	PBE + *U*_2_
Rh_19_–CO_t1_	3.50	3.60	–2.43	–2.39	–2.26
Rh_19_–CO_t2_	3.83	3.91	–2.39	–2.35	–2.21
Rh_19_–CO_t3_	5.33	5.48	**–2.45**	**–2.41**	**–2.28**
Rh_19_–CO_b1_	2.89	2.95	–2.40	–2.35	–2.20
Rh_19_–CO_b2_	3.00	3.05	→h2		
Rh_19_–CO_b3_	3.89	3.99	–2.39	–2.34	–2.15
Rh_19_–CO_b4_	4.22	4.33	–2.15	–2.10	–1.94
Rh_19_–CO_h1_	2.45	2.50	–2.21	–2.16	–2.00
Rh_19_–CO_h2_	3.18	3.26	–2.38	–2.34	–2.18
Rh_19_–CO_h3_	3.45	3.54	–2.32	–2.27	–2.11
Au_20_Rh_19_–CO_t1A_	4.33	4.37	–2.07	–2.03	–1.89
Au_20_Rh_19_–CO_t1B_	4.33	4.36	–1.93	–1.89	–1.75
Au_20_Rh_19_–CO_t2_	5.83	5.81	–2.03	–1.98	–1.84
Au_20_Rh_19_–CO_t3_	3.83	4.03	not converged		
Au_20_Rh_19_–CO_t4_	5.67	5.60	–2.02	–1.98	–1.84
Au_20_Rh_19_–CO_t5_	4.00	4.19	–1.03	–0.99	–0.84
Au_20_Rh_19_–CO_t6_	5.33	5.66	not converged		
Au_20_Rh_19_–CO_b1A_	4.44	4.42	–2.08	–2.03	–1.87
Au_20_Rh_19_–CO_b1B_	4.33	4.28	–1.97	–1.92	–1.76
Au_20_Rh_19_–CO_b2_	5.22	5.17	→h1A		
Au_20_Rh_19_–CO_b3_	4.67	4.81	→t2		
Au_20_Rh_19_–CO_b4_	3.44	3.62	–0.63	–0.58	–0.40
Au_20_Rh_19_–CO_b5_	5.22	5.13	→h1		
Au_20_Rh_19_–CO_b6_	5.00	5.14	→t4		
Au_20_Rh_19_–CO_b7_	4.11	4.22	→t4		
Au_20_Rh_19_–CO_h1A_	4.36	4.32	**–2.15**	**–2.10**	**–1.93**
Au_20_Rh_19_–CO_h1B_	4.36	4.28	–2.12	–2.06	–1.89
Au_20_Rh_19_–CO_h2_	4.09	4.21	→t2		
Au_20_Rh_19_–CO_h3_	4.00	4.11	→h1A		
Au_20_Rh_19_–CO_h4_	2.91	3.08	–0.41	–0.35	–0.15
Au_20_Rh_19_–CO_h5_	4.45	4.56	→b5 → h1		
Au_20_Rh_19_–CO_h6_	4.09	4.20	→t4		

a The most energetically favorable site for each NP is in bold.

**Table 3:** GCN and sGCN of Adsorption Sites, and Adsorption Energy, *E*_ad_ (eV), of a CO Molecule on Au_36_Rh_19_ and Au_52_Rh_19_ at the PBE, PBE + *U*_1_, and PBE + *U*_2_ Level of Calculation, as Shown in Table 2 ^a^

site	GCN	sGCN	PBE	PBE + *U*_1_	PBE + *U*_2_
Au_36_Rh_19_–CO_t1_	4.33	4.34	–1.97	–1.93	–1.79
Au_36_Rh_19_–CO_t2_	5.67	5.60	–2.00	–1.96	–1.82
Au_36_Rh_19_–CO_t3_	4.33	4.52	–0.61	–0.56	–0.42
Au_36_Rh_19_–CO_t4_	5.67	5.98	–1.08	–1.03	–0.89
Au_36_Rh_19_–CO_t8(7)_	5.67	5.89	–0.72	–0.68	–0.54
Au_36_Rh_19_–CO_t9(6)_	4.33	4.47	–1.22	–1.18	–1.04
Au_36_Rh_19_–CO_b2_	5.22	5.13	→h1		
Au_36_Rh_19_–CO_b3_	5.22	5.34	→h1		
Au_36_Rh_19_–CO_b4_	4.33	4.43	→t2		
Au_36_Rh_19_–CO_b5_	4.33	4.55	→t2		
Au_36_Rh_19_–CO_b7_	4.33	4.52	–0.59	–0.54	–0.37
Au_36_Rh_19_–CO_b14(8,10,13)_	5.22	5.44	→h10		
Au_36_Rh_19_–CO_h1_	4.36	4.28	**–2.08**	**-2.03**	**-1.86**
Au_36_Rh_19_–CO_h2_	4.64	4.73	→h1		
Au_36_Rh_19_–CO_h3_	4.36	4.46	→t2		
Au_36_Rh_19_–CO_h9(11)_	4.64	4.82	–0.08	–0.03	>0
Au_36_Rh_19_–CO_h10(6,7,8)_	4.36	4.54	–0.21	–0.15	>0
Au_52_Rh_19_–CO_t1(3)_	4.33	4.47	**–1.12**	**–1.08**	**–0.94**
Au_52_Rh_19_–CO_t2(4)_	5.67	5.89	–0.73	–0.68	–0.55
Au_52_Rh_19_–CO_t5_	5.83	6.05	–0.66	–0.62	–0.48
Au_52_Rh_19_–CO_b5(1,3)_	4.33	4.49	→t4		
Au_52_Rh_19_–CO_b8_	5.44	5.65	→b10		
Au_52_Rh_19_–CO_b10_	6.00	6.25	–0.34	–0.28	–0.11
Au_52_Rh_19_–CO_h1_	4.36	4.53	–0.18	–0.12	>0
Au_52_Rh_19_–CO_h7_	5.27	5.49	→b10		

a The most energetically favorable site for each NP is in bold.

There are ten nonequivalent sites on Rh_19_, among which three are top sites. They correspond to CO binding at a Rh 5-fold vertex (t1), to the eight-coordinated Rh of the central pentagonal ring (t3), and the 5-fold Rh in the other pentagonal ring (t2). There are two t1, five t3, and ten t2. The bridge sites are ten of type b1; b2 labels one of the ten bridges between Rh, belonging to a pentagon close to a vertex; five b4 bridges and two Rh in the central ring. b3 labels the site between a Rh of a pentagon and a neighboring Rh of the central ring. There are ten h1 sites close to the vertex. h2 and h3 are between the pentagonal rings, and we count ten of each. Those adsorption sites represent a complete characterization of the NP surface. PBE and PBE + *U*s levels predict b2 as an unstable site with the CO molecule moving to a nearby h2. The t3 site is the most energetically favorable for CO adsorption on Rh_19_, with t1, b3, and h2 energetically close, differing by 0.08 eV. Our results agree with experimental data that find CO favorably adsorbed on various sites,^30,32,35^ with the top being slightly favorable. Further, CO binds about 0.4 eV more strongly on Rh_19_ than on Rh(111).

Regarding the RhAu NAs, the number of nonequivalent sites varies with Au-loading: 27 on Au_20_Rh_19_, 24 on Au_36_Rh_19_, and 17 for Au_52_Rh_19_. The number of sites with a mixed chemical environment is 15 and 9 on Au_20_Rh_19_ and Au_36_Rh_19_, respectively. The first addition of gold atoms affects the stability of some sites and, in general, increases the sGCN of the sites. This also relates to the structural change of the Rh structure from a double-Ih to a Dh-Ih. For that reason, we expect a reduction in the strength of the CO bonds with Rh, as seen in Figure 4 and Table 2. The further addition of Au does not change the sGCN further, and as a consequence, the adsorption energy varies little. Sites along the Au/Rh step, mainly bridge and hollow, result in stability. Sites surrounded only by Au atoms are expected to bind the CO molecule more weakly due to gold’s chemical inertness. At the same time, the presence of Rh stabilizes the adsorption of CO on Au-sites, even more than other Au-alloys, as ∼0.22 eV on Au@Cu-NPs,^95^ whereas AuRh is stabilized by ∼0.5 eV, similarly to AuPt.^96^

**Figure 4:**
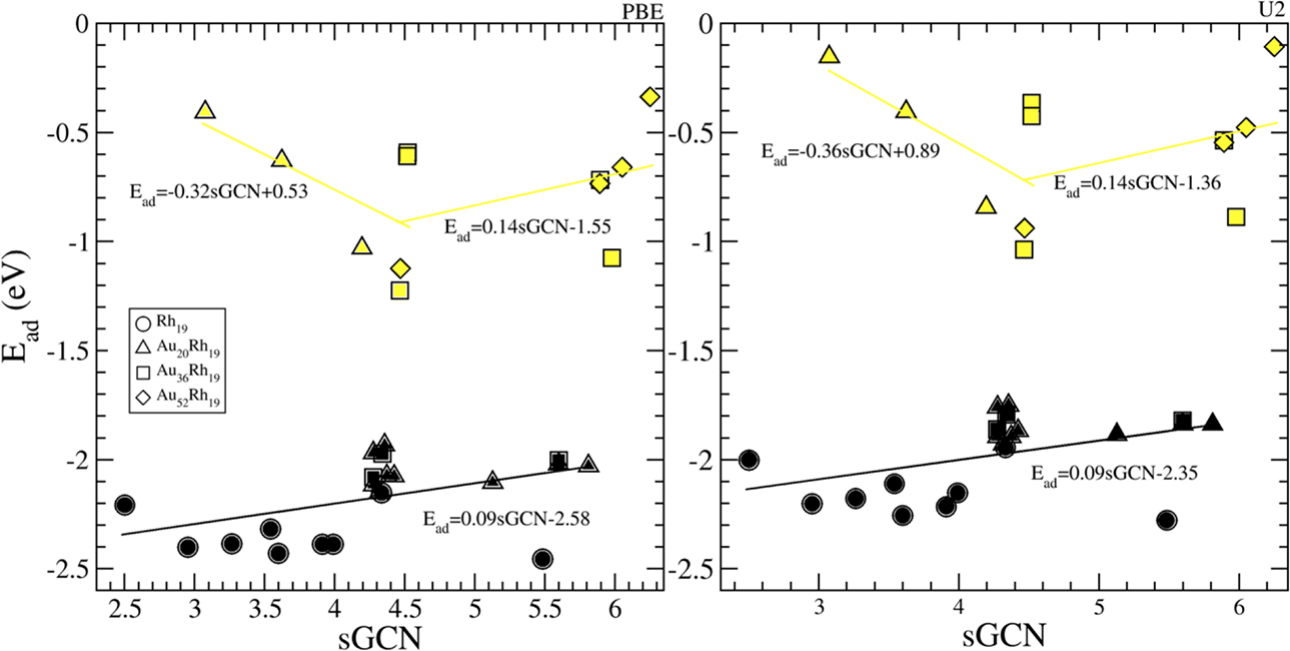
Adsorption energies (*E*_ad_) of CO on Rh-based nanoparticles as a function of the strained generalized coordination number (sGCN). Dark-black symbols refer to a site with a Rh chemical environment, while light-yellow symbols refer to a Au chemical environment. Results from PBE and PBE + *U*_2_ simulations are presented on the left and right, respectively. Linear regression lines for rhodium (dark-black) and gold (light-yellow) are provided.

On Au_20_Rh_19_, CO binds preferentially on a hollow site (site h1A), one of the five Rh 3-fold hollow sites near the 5-fold vertex on the A-side. However, h1B and t1A are within 0.08 eV of h1A. In general, at the PBE + *U*_2_ calculation level, the energy difference between the top and hollow most stable adsorption sites is reduced in this NA, compared to the pure Rh systems, Rh_19_ and Rh(111). The two Au/Rh steps of the scarf decoration differ. On the A-side, there are five 4-fold hollow sites (h3) made by two Rh and two Au atoms. Nevertheless, they are unstable, and CO moves to h1A. Furthermore, sites on the A-side (Figure 3) tend to bind CO strongly. There is a difference of 0.14 eV between t1A and t1B, 0.11 eV between b1A and b1B, and 0.03 eV between h1A and h1B. Although these sites have similar generalized coordination numbers and charge redistribution, the length of the CO bond is longer on t1B and b1B than on their corresponding sites on side A. We emphasize that specific sites, such as t3 and t6 on Au_20_Rh_19_, do not converge to a reliable electronic density. Moreover, half of the bridge sites accessible with at least a Rh atom -namely, b2 and b3 on side A and b6 and b7 on side B– are unstable. Similarly, more than half of the hollow sites are also unstable. Due to the instability of several sites, t4 and t2 are likely to bind CO alongside h1A, t1A, and t1B. For instance, t4, where CO binds to an Rh with three neighboring Au, could play a role as the other three sites converge to it, although it binds 0.13 eV less than h1A. For Au-rich sites, t5, b4, and h4 of Au_20_Rh_19_, *E*_ad_ ranges between −1.03 (−0.84) and −0.41(−0.15) eV at PBE (PBE + *U*_2_), showing a positive effect of Rh on Au. Notably, t5 holds remarkable stability, comparable with Pt and better than AuPd or AuCu.^95^

On Au_36_Rh_19_, the Au-cup is delimited by a pentagonal Au/Rh step with three Au-atoms along the edge, covering the Rh-Ih_13_ and leaving just six atoms of Rh at the surface (side B in Figure 3). Those six Rh-atoms form one atop (t1 site, with sGCN = 4.34), five bridge sites (b1), and five hollow sites (h1). The most stable site is h1, but t1 is just 0.07 eV from it. This result is similar to that obtained on Au_20_Rh_19_ and indicates that the Au decoration destabilizes the atop adsorption in favor of the hollow one. The b2 adsorption site, formed between two surface Rh atoms and with Au atoms in their neighborhood, is obtained as unstable and converges toward an h1 site along the calculations. There are five midcoordinated sites (t4) along the Au/Rh step and a more coordinated top Au-site above the 5-fold vertex of the Rh-Ih_13_ (site t9, with sGCN = 5.89). On the t2 adsorption site, the CO is atop one of the Rh atoms facing the Au-cup, and this site is 0.04 eV from the most stable. The t9 atop site (with sGCN = 4.47) is the most stable CO adsorption site on Au. This site is a 5-fold vertex, sitting above the Rh-vertex of an Ih_13_, showing again the importance of a 5-fold structure to enhance the CO-binding on Au. Notably, the top site on the Au-edge facing the Rh (site t4, with sGCN = 5.98) is reasonably stable, with an adsorption energy of 0.15 eV from the most stable t9.

On Au_52_Rh_19_, the landscape for CO-binding offers a variety where the molecule binds on Au-anchors. The sites are all rather coordinated from 4.47 to 6.25. The sites on the Au-chiral rings form several hollow sites and six bridge sites. Those sites weakly bind CO despite their sGCN, and some of them are not stable. Two t1 sites (sGCN = 4.47), located above the Rh 5-fold vertex of the Rh-dIh_19_, are the energetically more favorable with an *E*_ad_ of −0.94 eV, with a tiny reduction regarding their counterpart site on the ball-cup. Comparing the Au_36_Rh_19_ with the Au_52_Rh_19_ at the PBE­(PBE+U2) level, the most energetically favorable Au site shows that the adsorption energy goes from −1.22(−1.04) to −0.59(−0.37) eV in the first and from −1.12(−0.94) to −0.34(−0.11) eV in the later, respectively. CO on Au binds strongly on top of the 5-fold vertex (t1, sGCN = 4.47), confirming the beneficial effect of Rh on Au-5-fold atop sites.

Figure 4 shows the structure-adsorption relationship in terms of the sGCN. We note that adsorption on Rh-rich sites weakly depends on the sGCN, with a slope of 0.09, regardless of the *U*-value applied. Between PBE and PBE + *U*_2_, the intercept shifts by 0.23 eV. On the other hand, Au-rich sites show an interesting nonlinear behavior, with peaks at sites with midcoordination (sGCN = 4.7). The left side has a negative slope of −0.32, while the second arm is 0.14 (PBE), which is in line with the prediction on Au(111).^95^ At the PBE + *U*_2_, the slopes are −0.36 and 0.14 for the left and right arm, respectively.

Figure 5a maps the charge redistribution and provides some geometrical features for CO adsorbed on the most stable sites of the NAs. Additionally, Figure S12, in the Supporting Information, contains the complete list of geometrical features and charge redistribution for CO adsorbed on all sites of Rh_19_ and Au_20_Rh_19_, while Figure S13 for Au_36_Rh_19_ and Au_52_Rh_19_. Regarding the α angle, CO relaxes almost perpendicular to the NP’s edges and facets in all cases. For the top adsorption mode, Rh–C–O forms an almost flat 180° angle. The main deviation, about 5%, is for t5 on Au_20_Rh_19_. The *d*_CO_ bond elongation follows the same trend as on Rh(111), with *d*_CO_ decreasing as hollow → bridge → top. For top sites adsorbed on gold, the *d*_CO_ is elongated between 0.02 and 0.03 Å, and for top sites adsorbed on rhodium, the *d*_CO_ is elongated by 0.01 Å. The longer *d*_CO_ bond length on Au could be a relevant piece of information regarding the CO bond activation in catalysis. Moreover, the longest stretch is for hollow sites and b4 on Rh_19_. These findings align with numerical estimates of CO on a cuboctahedral Rh_55_ ^35^ and for truncated octahedra with *N* = 38.^40^ Here, *d*_M–C_ is between 1.8 and 2.10 Å.

**Figure 5:**
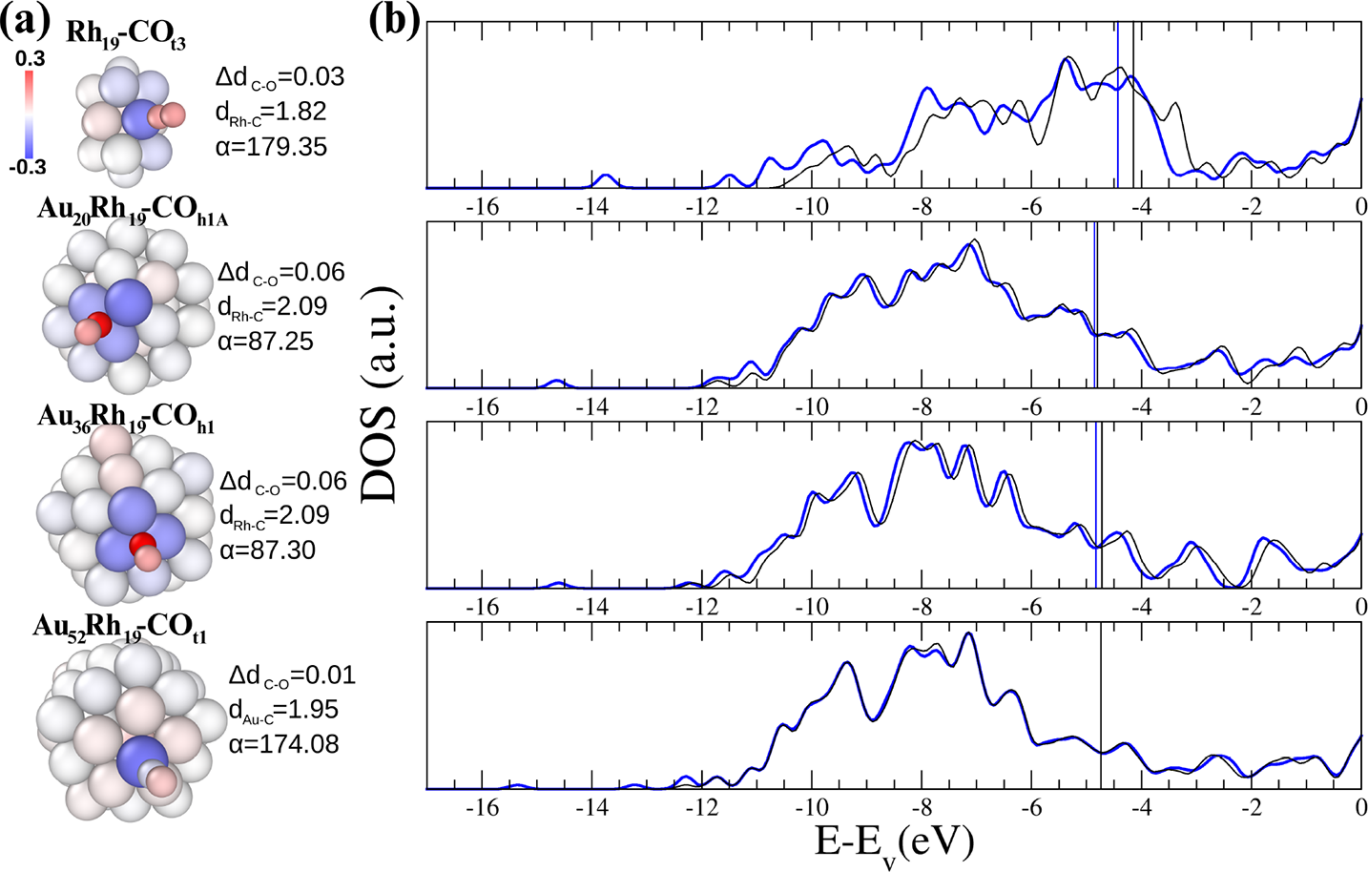
Panel (a): Charge redistribution and geometrical descriptors for the most energetically favorable site on Rh_19_, Au_20_Rh_19_, Au_36_Rh_19_, and Au_52_Rh_19_. Atoms are colored according to their charge redistribution (Δ*q* ^*i*^ in units of *e*, as shown in eq 2). Blue indicates electron depletion, and red indicates electron acquisition. The geometrical descriptors Δ*d*_CO_ and *d*_M–C_, with M = Au and Rh, are the CO bond length difference between the adsorbed and isolated molecule and the average metal-CO distance after adsorption, respectively (in Å). α indicates the angle between the CO axis and the plane containing the Rh/Au-anchoring C-atom. (b) Corresponding DOS profile before (dark-black) and after (light-blue) CO adsorption at PBE + *U*_2_. The vacuum level is set at zero, and the vertical lines refer to the Fermi level of each system.

Regarding the Δ*q* results presented in Figure 5a, the lighter red color and lower charge gain of C on top sites than on hollow sites are consistent with the stronger metal *d* → CO 2π* back-donation on hollow sites. The O atom is always negatively charged (red color), while C receives electrons from the substrate on Rh sites when on smaller NAs, even more than the O atom. On the other hand, on Au_52_Rh_19_, only O is negatively charged. For CO on Au_20_Rh_19_ and Au_36_Rh_19_, Rh atoms donate most of the charge, while Au atoms are mildly affected in this sense. On sites with a rich Au-chemical environment, e.g., t5, b4, h4 on Au_20_Rh_19_, C and O atoms are less negatively charged. For example, on t5 of Au_20_Rh_19_, t3, t4, t8, t9 of Au_36_Rh_19_ and t1, t2 and t5 of Au_52_Rh_19_, C is not receiving any electrons. Consequently, there is a negligible stretch in the CO bond length. The 5-fold top usually localizes the charge distribution better than all other sites. t3 on Rh_19_ shows a more delocalized charge redistribution among Rh atoms due to the intrinsic symmetry of the dIh. The charge transfer tends to be greater on hollow sites, with C receiving about 0.35*e* from Rh and O about 0.2*e*. Considering that these sites show a longer C–O bond and focusing on the point that for all nanoparticles the h1 site is the one that elongates the molecule the most, this indicates that hollow sites could be more active for CO catalysis on Rh-NAs, especially along reduction reactions. The longer *d*_CO_ bond elongation observed on hollow sites compared to top can be explained in terms of metal *d* → CO 2π* backdonation, which is more intense in hollow sites. A similar effect is also reported for other smaller molecules.^97,98^ In addition, a reduction of the CO-Au_20_Rh_19_ electron donation^74^ is seen for CO adsorbed on a site formed by Au atoms, affecting the CO stability compared to Rh_19_. Regarding all evaluated positions, there is a negligible correlation between the charge redistribution and the adsorption energy, as discussed in Supporting Information, Figures S14 and S15.

Panel (b) of Figure 5 reports the DOS before and after CO-adsorption on the most energetically favorable sites only. In addition, Figure S10 in the Supporting Information shows that the *U*_2_ correction has very little effect on the DOS of the same systems, while Figure S11 in the Supporting Information shows the pDOS magnification of the CO p-states for the most stable top (t3) and hollow (h2) CO adsorption sites on Rh_19_, confirming that 4d-2π* backdonation is stronger for CO at the hollow site than at the top site. For pristine NAs, the work function rises from Rh_19_ to the Au-scarf decoration, remaining approximately at 5.5 eV for various Au loadings. The addition of CO dramatically impacts the DOS of Rh_19_, increasing its work function and indicating significant orbital mixing, as evidenced by modifications in the peaks. These changes contribute to strong CO binding. The CO effect is less visible in NAs because these systems have more valence electrons than the (2 × 2 × 4)-Rh(111). Nonetheless, minor differences between dark-black (pristine NA) and light-blue (after adsorption) lines still indicate mixing between the CO orbitals and the AuRh *sd*-band. The main changes occur across the Fermi level, between −6 and −7, −12 and below −14 eV, as in the case of the CO adsorption on the Rh(111) slab. The DOS analysis indicates that the interaction between CO molecular orbitals and the nanosystems mirrors that seen on the Rh(111) slab. We emphasize the importance of utilizing a computational method that effectively addresses the 2π*-4*d* backdonation issue.

## Conclusions

We present a comprehensive study of CO adsorption on Rh(111), a double-icosahedron Rh_19_, and on three decorations with Au, from a scarf of twenty-Au atoms to an entire chiral gold shell. The mapping of adsorption sites on Rh-based nanoalloys combines the strained generalized coordination number (sGCN) and the local chemical environment of each site, simply the number of Au and Rh atoms surrounding it. We employ DFT simulations at the PBE and PBE+U levels, and the CO-adsorption energy increases linearly with U with a slope that is site-dependent. A *U* as small as 0.65 eV is enough to retrieve the correct adsorption stability order (top → low → bridge, from the most stable to the least stable) for CO on Rh(111). A further increment of the *U*-value to 2.6 eV enables us to predict the adsorption energy on Rh(111) in agreement with experiments and enlarges the top preference to 40 meV compared with the hollow site.

As expected, the level of CO stabilization on Rh_19_ is greater, with an average increment of about 0.6 eV to Rh(111). The top 5-fold vertex is the energetically preferred site on Rh_19_, in line with the adsorption on the Rh(111) extended surface. Notably, the top energetic preference over the hollow sites increases from only 70 to 200 meV when using a *U*-value of 2.6 eV. Consequently, the energetic preference of CO on the top adsorption site reported on Rh(111) is reinforced on the Rh_19_ nanoparticle. Indeed, the slope of the sGCN adsorption-energy profile on Rh-sites is as tiny as 0.09 eV, suggesting that adsorption can occur in several modes, in agreement with experimental data.

The addition of Au atoms increases the number of possible sites. Interestingly, CO adsorbs preferentially on hollow sites when adsorbed on an Rh anchor on AuRh nanoalloys. Nonetheless, mixed Au and Rh sites do not bind CO, but CO preferentially sits onto hollow sites close to the 5-fold vertex. As believed, the presence of Au lowers the adsorption energy on Rh, but this is independent of the loading. On the other hand, Rh eventually enhances Au’s ability to react with CO because Rh impedes any structural rearrangements within Au-decorations. Further, CO binds atop an Au atom along the Au/Rh step or on a 5-fold vertex with an Rh beneath Au. The profile of adsorption energy on Au-sites according to the sGCN is nonlinear and shows a peak at midcoordinated sites, with sGCN at 4.47. The profile at higher coordination is similar to that of CO on Au-pure nanoparticles, but the low-coordinated sites exhibit peculiar behavior. The strong atop adsorption on Au’s 5-fold vertex, about −1 eV, makes AuRh a promising nanoalloy. The present study sheds light on the CO adsorption on Rh nanoparticles and AuRh nanoalloys, revealing that the Hubbard correction effectively describes these systems, as observed on the Rh(111) surface.

## Supplementary Material



## Notes

Isolated CO, Rh(111), and Rh-based nanosystems; dependency of CO DOS on the *U* value; dependency of adsorption energy versus *U* for top and hollow sites, pDOS of Rh(111) and of top and hcp-hollow adsorbed CO on Rh(111); comparison between DOS calculated with different *U* values; results on the electronic structure obtained at the NSCF@(PBE + *U*) and PBE + *D*_3_ levels; discussion of the induced geometrical strain on Rh(111) and results obtained on the (2 × 2 × 6) slab; mapping of the sites of Rh-naosystems in terms of their coordination and generalized coordination plus considerations about the impact of U on adsorption energies and magnetization for the Rh-based nanosystems; comparison between nanosystem DOSs at PBE and PBE + *U*_2_ levels; full characterization of charge redistribution and the geometrical parameters for CO adsorbed on Rh_19_ and Au_20_Rh_19_; and graph showing the correlation between the CO acquired charge with the adsorption energy (PDF)
