# Supplementary material for: Gold Decoration To Improve Rh-Nanoalloys for CO-Adsorption
Source: J Phys Chem C Nanomater Interfaces. 2025 Apr 24;129(24):10854–65. doi: 10.1021/acs.jpcc.4c08091 (PMC12186612; doi:10.1021/acs.jpcc.4c08091)
Supplement: Supplementary file 1 [file jp4c08091_si_001.pdf]

Supporting Information: A gold decoration to improve Rh-nanoalloys for CO-adsorption

Letícia F. Basso,† Mirko Vanzan,† L. Prati,‡ Vagner A. Rigo,†,¶ and Francesca Baletto*,†

†*Department of Physics, University of Milan, Milan, 20133, Italy*

‡*Department of Chemistry, University of Milan, Milan, 20133, Italy*

¶*Department of Natural Sciences, Federal University of Technology - Paraná, Cornélio Procópio, 86300-000, Brazil*

E-mail: francesca.baletto@unimi.it

Here, we collect additional and complementary information to the main text. These Supporting Information are separated into three chapters, the first containing information on the free CO molecule, the second on Rh(111) surface and the third on Rh-based nanoparticles.

Results on CO isolated

Figure S1 shows the DOS and pDOS of the isolated CO molecule calculated at the PBE and the PBE+U2 (U = 2.6 eV) levels. The molecular structure slightly changes between the two methods and clearly shows the typical CO electronic configuration, with a double-degenerate 1π and $2\pi^*$ peaks, and the 5σ which are the key orbitals in CO-metals interactions.[1,2]

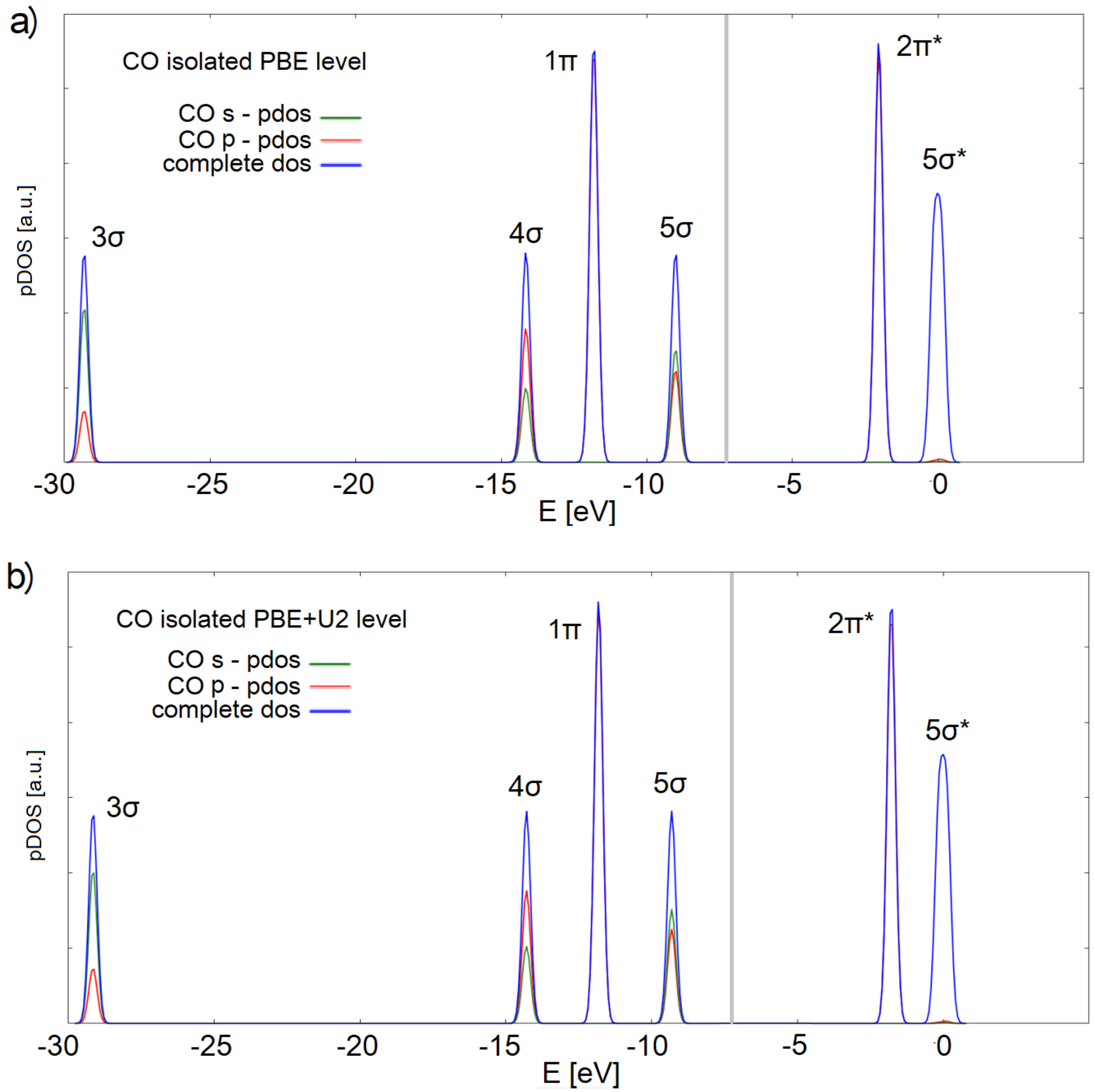

Fig. S1: DOS and pDOS for the isolated CO molecule obtained at the a) PBE level and b) PBE+U2 level. Zero energy represents the vacuum energy, and the grey line marks the Fermi energy.

Results on the Rh(111) surface

The starting point for this work was the observation of how the Hubbard correction, with the standard scheme from Dudarev,[3,4] acts in the Rh(111) system with the adsorbed CO molecule. Figure S2 shows how the adsorption energy changes with respect to the applied U

in the case of top, bridge and hcp hollow sites. We observe a crossing between the top and hcp lines, with the top becoming more stable than the hcp hollow for U larger than 0.3 eV. As reasonable U values, we select U1 = 0.65 eV as it provides an energy difference of 10 meV between the two sites, and U = 2.6 eV, which provides a top adsorption energy compatible with what was observed in the experiments.[5,6]

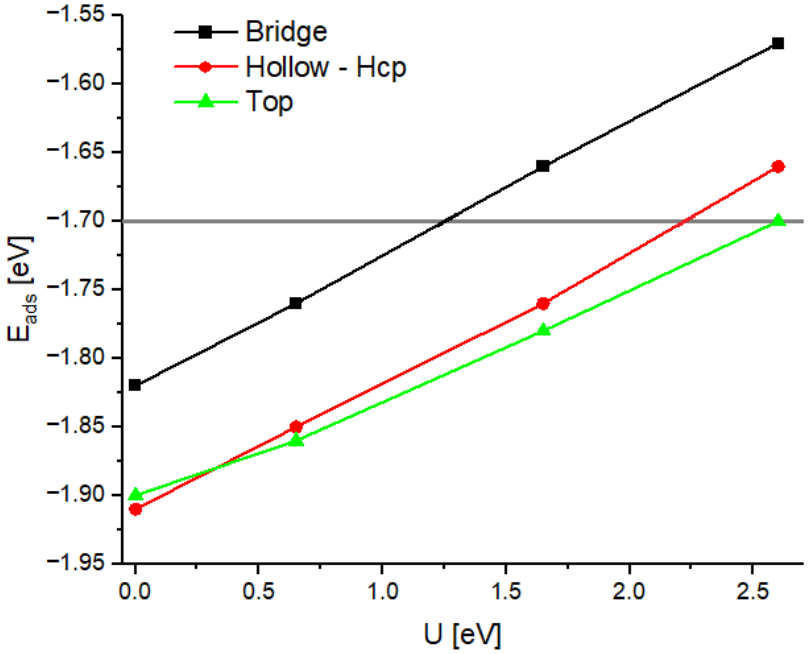

Fig. S2: Variation of the CO adsorption energy on Rh(111) in the case of a 2×2×4 slab. Calculations made at U = 0, 0.65, 1.65 and 2.6 eV. Lines are a guide for the eye and do not represent a linear fit. The grey line marks the -1.70 eV level, the experimental value for top adsorption.

Particularly, we consider the CO relaxed at the PBE level on the 2×2×4 slab. We calculated the PBE+U electron density, and then we performed a non-self-consistent calculation at the PBE level using the PBE+U charge density.

Figure S3 shows the DOS and pDOS of the pristine Rh(111) surface. As expected, the valence bands are made primarily by Rh d-orbitals with a minor contribution from the s-orbitals. Rh p-orbitals do not sensibly contribute to any of the frontier bands.

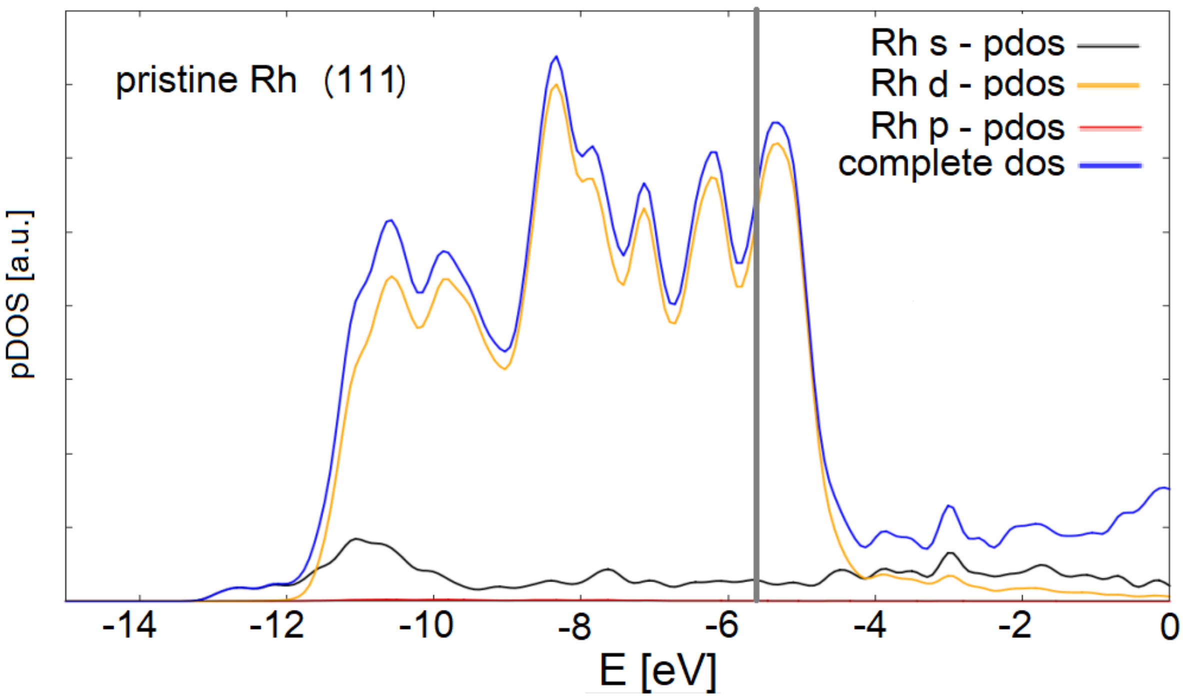

Fig. S3: At PBE level, DOS and pDOS of the pristine Rh(111) 2×2×4 slab. Zero energy represents the vacuum energy, and grey lines mark the Fermi energy.

Figure S4 show the DOS and pDOS of the CO-Rh(111) system with CO adsorbed on a) top and b) hollow - hcp sites, calculated at the PBE level. As observed, CO significantly contributes to the peak around -15 eV through the 4σ orbital and to the spectral maxima near -12 eV via the 1π orbital. A minor contribution from the 5σ orbital is also noticeable around -8 eV. Near the Fermi energy, a small but nonzero contribution from the CO $2\pi^*$ orbital is present, indicating $2\pi^*$ to d back-donation. This effect is known to overestimate the CO adsorption energy in PBE calculations, a well-documented phenomenon in the literature.[1,2]

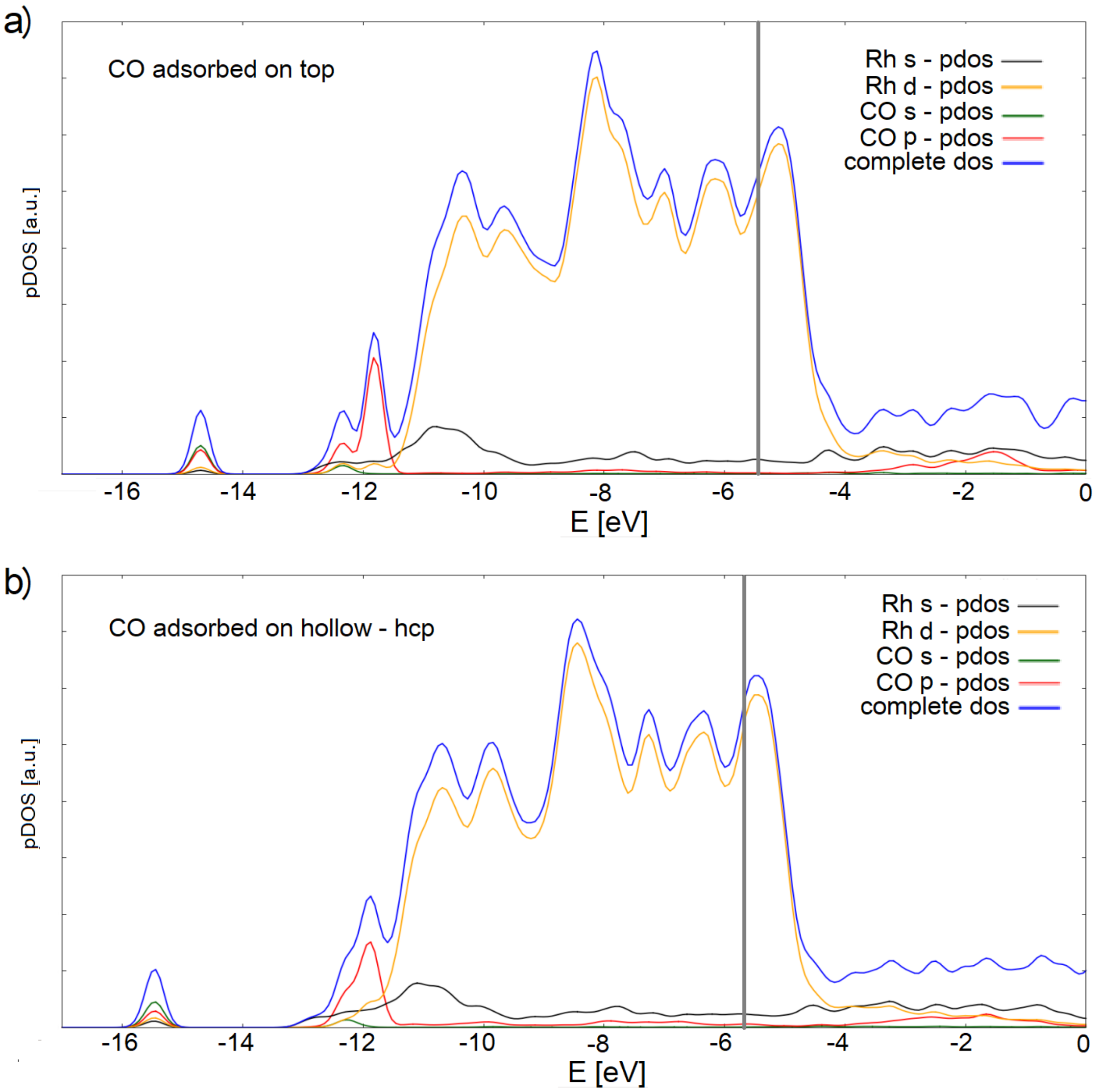

Fig. S4: At PBE+U2 level, DOS and pDOS of the pristine CO-Rh(111) system with CO adsorbed on a) top and b) hollow-hcp. Zero energy represents the vacuum energy, and the grey lines mark the Fermi energy.

Figure S5 shows how the choice of U modifies the DOS profile. As noticeable the shapes are very similar between PBE, PBE+U1, and PBE+U2. The only notable difference is the small rigid shift involving the plots. This came from the fact that the different systems show a slightly different vacuum energy. As the simulation cell has a finite size along the direction perpendicular to the surface, the vacuum level is not zero and depends on the

electronic density, which in turn depends on U. Apart from this rigid shift, the plots are basically identical. Figure S6 shows the pDOS magnification of CO orbitals at the PBE and PBE+U2 levels for CO on the top adsorption site. The magnification focuses on the main differences between PBE and PBE+U2, where more intense peaks are noted at PBE levels (red curve), compared to PBE+U2 (blue curve), confirming a reduction in the Rh 4d$\rightarrow\pi^*$ backdonation using the PBE+U2 corrected functional.

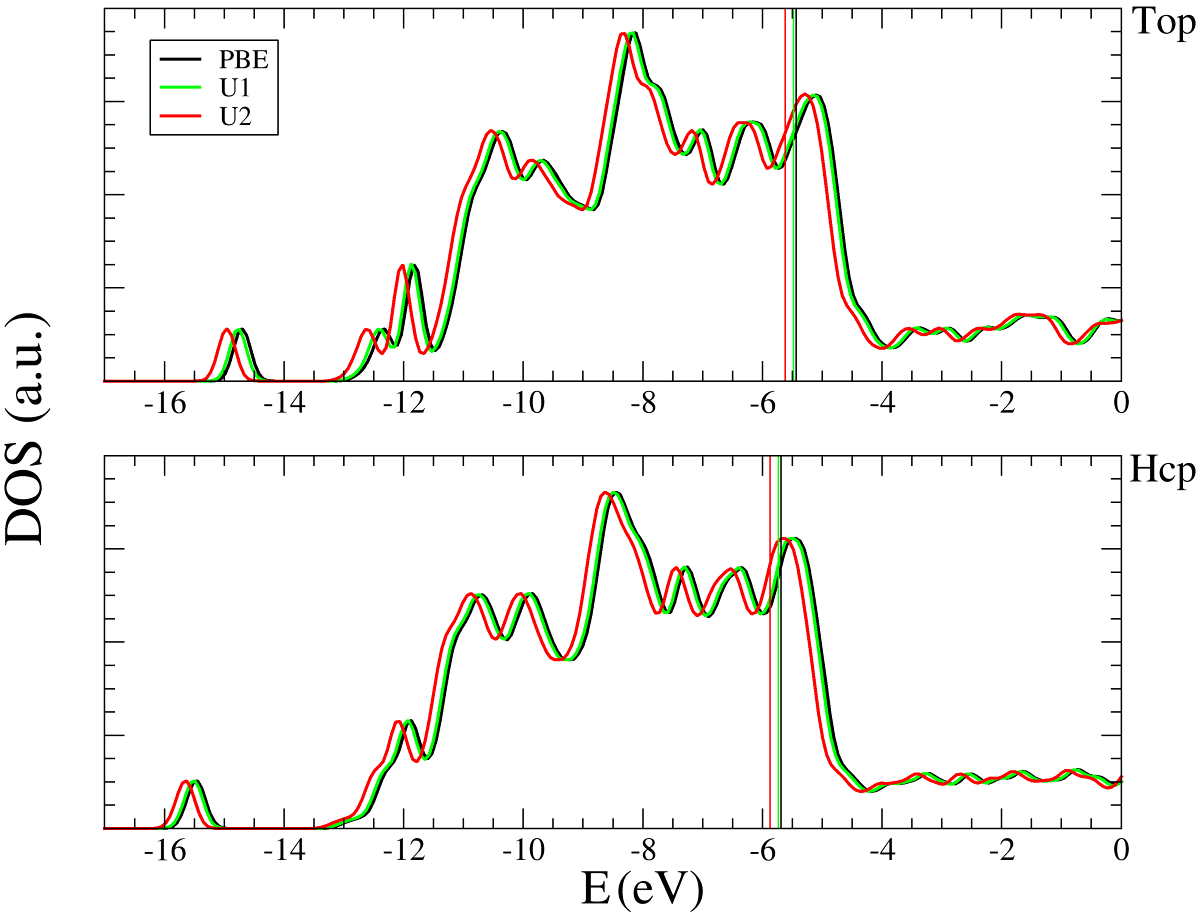

Fig. S5: Effect of U on the CO-Rh(111) systems. Zero energy represents the vacuum energy, vertical lines mark the Fermi energy.

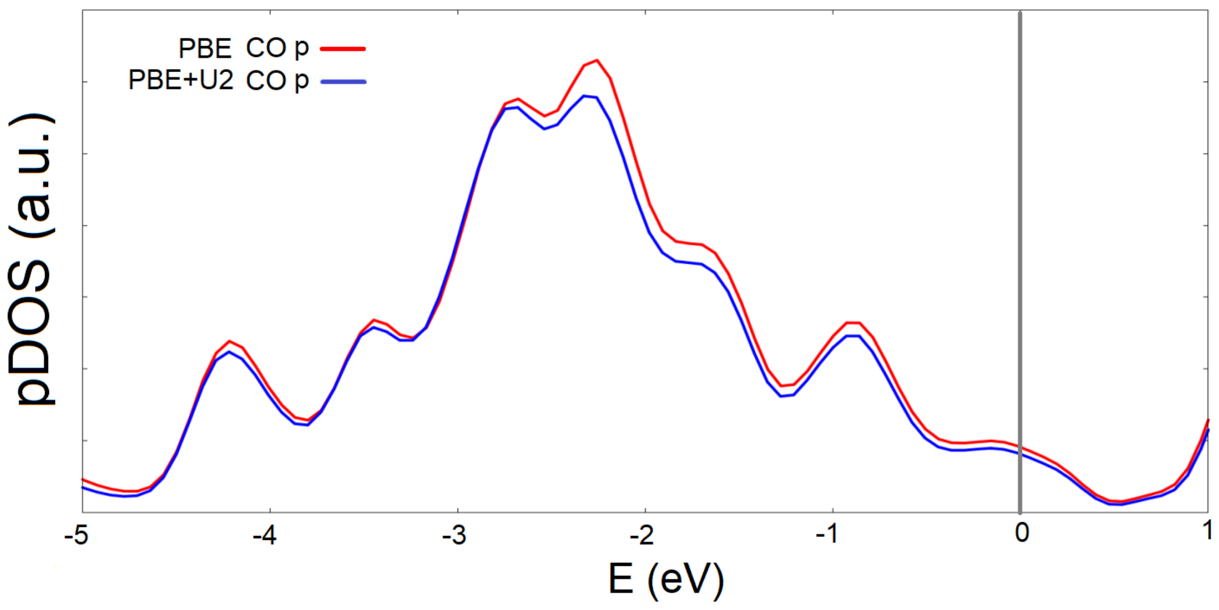

Fig. S6: pDOS of CO p-orbitals when the CO molecule is on top adsorption mode on Rh(111) extended surface at PBE and PBE+U2 levels of calculations. The Fermi level is at zero energy.

Other correction to PBE

Non-self-consistent calculations at the PBE level using the charge density obtained at the PBE+U level have been also applied, as proposed by Patra *et al.*,[7] named as NSCF@(PBE+U) procedure. Here non-self-consistent PBE calculations are made using a U-corrected charge density. This method has been adopted by Patra *et al.* to evaluate the CO adsorption on Pt(111). The NSCF@(PBE+U) methodology does not improve the adsorption energy. Specifically, with U = 0.65 eV, the calculated adsorption energy remains -1.90 eV for both the top and hcp-hollow sites on Rh(111). Moreover, we verified that the DOS and pDOS of CO adsorbed on the top, hcp-hollow, fcc-hollow and bridge sites on the Rh(111) 2×2×4 surface do not sensibly change compared to the DOS/pDOS calculated at the PBE level (not shown here). Consequently, we have decided not to pursue this approach further for CO interactions with Rh-based nanosystems.

We tested the addition of the van der Waals correction to PBE (PBE+D3).[8] In fact, dis-

persive forces should be included as they play a role in determining the site preference for CO on Pt(111).[9,10] At the same time, Illas and coworkers claimed that including dispersion forces overestimates the experimental values of CO binding to the metal.[11] For CO on Rh(111), PBE+D3 increases the adsorption energy but fails in reproducing the correct energetic order between the adsorption modes, as it stabilizes hollow sites by an extra 22 meV compared to PBE. PBE+D3, being detrimental in reproducing the experimental results[12] and the correct energetic order between CO adsorption positions on Rh(111), is not considered further.

Slab size and strain effects

Table S1 contains the height difference between the layers of the 2×2×4 Rh(111), after and before CO adsorption. We can conclude that the Rh(111) subsurface layer is not significantly affected by the presence of CO.

Tab. S1: Difference between interlayer distances in the 2×2×4 Rh(111) system (Δz), after and before the CO adsorption. Here, layer 1 has all the atoms fixed at the bulk value and layer 4 is the surface. Calculations are performed at the PBE level.

Layer	Top (Å)	Hcp (Å)	Fcc (Å)	Bridge (Å)
1-2	-0.003	-0.005	-0.004	-0.003
2-3	0.014	-0.013	-0.008	0.001
3-4	0.036	0.051	0.054	0.048

The CO adsorption energy on surfaces can be written as the sum of two contributions, $\mathrm{E_{ad}} = \mathrm{E^{c}_{ad}} + \mathrm{E^{s}_{ad}}$, one chemical E^{c}_{ad}, and the other due to the geometrical rearrangement of the atoms E^{s}_{ad}. E^{s}_{ad} is the total energy difference between the fully-relaxed Rh(111) and the one where Rh-atoms keep their position after adsorption. The strain contribution is much smaller than the chemical one, but it could have a role in ordering the adsorption modes. So, E^{c}_{ad} on top is -1.84 and on hcp-hollow is -1.82 eV. This result indicates that the surface strain contains a small but not zero contribution to the E_{ad}, and the top site presents the lowest E^{c}_{ad}.

Figure S7 represents the displacement of surface Rh atoms with respect to the pristine

surface, on the z axes. As visible top adsorption leads to the highest displacement, followed by bridge and then hollow sites.

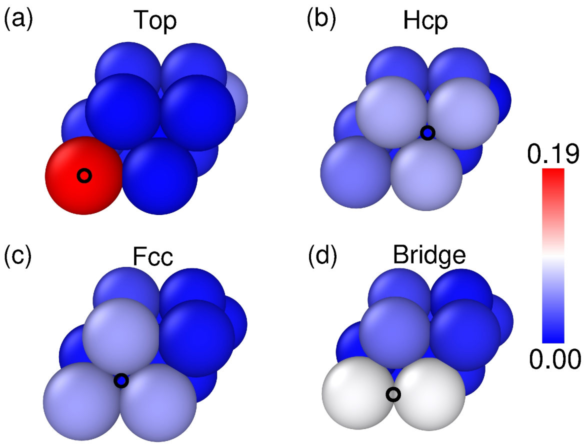

Fig. S7: Displacement map of Rh-atoms with respect to their z position before CO-adsorption, for (a) top, (b) hcp hollow, (c) fcc hollow, and (d) bridge sites. We report a 2×2×4 Rh(111) slab. Black circles represent the position of the adsorbed CO molecule in each geometry. The scale is in Å units. We only have positive displacements hence elongation of the Rh distances.

Table S2 show the results obtained in the case of the 2×2×6 Rh(111) slab.

Tab. S2: Adsorption energy, E_{ad}, and notable structural descriptors for the CO-Rh(111) 2×2×6 system. calculated at the PBE level.

Site	E_{ad}	Δd_{C-O}	d_{Rh-C}	Δd_{Rh-Rh}	Δz
@PBE 2×2×6					
Top	-1.82	0.02	1.83	0.002	0.030
Hcp	**-1.89**	0.05	2.09	0.027	0.035
Fcc	-1.78	0.05	2.10	0.008	0.037
Bridge	-1.78	0.04	2.01	0.014	0.055

Results on Rh-based nanoparticles

To understand better the CO interaction with Rh_{19}, $Au_{20}Rh_{19}$, $Au_{36}Rh_{19}$ and $Au_{52}Rh_{19}$, we took a closer look at the coordination number (CN), the generalized coordination number (GCN) and the strained GCN (sGCN). The nominal CN of the ith-atom is simply the count of how many atoms fall within a certain cutoff from the atom i. Here the cutoff is set

at 3.08 Å. The GCN of a site α is calculated accordingly to the Calle-Vallejo's works,[13,14] and reads:

$$GCN(\alpha) = \frac{\sum_{j=1} CN(j)}{cn_{max}}, \quad (1)$$

with α representing the site and with j running over the metallic atoms forming the site. cn_{max} is the bulk coordination equals to 12, 18, 22 and 26 for top, bridge, three-fold and four-fold hollow modes respectively.

To take into account the local distortions likely to be present on nanoparticles and the presence of two chemical species, we calculate the strained GCN ($sGCN$)[13,14] as:

$$sGCN(\alpha) = \sum_{i \in neighboursite} \sum_{j \in NN(i)} \frac{d_{ij}^{bulk}}{d_{ij}}, \quad (2)$$

where i are the metallic atoms binding the molecule, and j are the nearest neighbor of atom i, lying at a distance d_{ij}. d_{ij}^{bulk} is the pair distance between two atoms i and j from the bulk; this value varies depending on the site observed. When the molecule is adsorbed on rhodium d_{Rh-Rh}^{bulk} = 2.710 Å and when adsorbed on gold d_{Au-Au}^{bulk} = 2.939 Å and for sites between rhodium and gold the value is the average between the bulk of both chemical elements; d_{Au-Rh}^{bulk} = 2.825Å. The sum in the equation runs over their neighbors. Shared neighbors are considered the only ones.

The $sGCN$ values for atop sites are shown in Figure S8. The $sGCN$ and the GCN is calculated using SNOW, available at *github.com/nanoMLMS/pySNOW*.

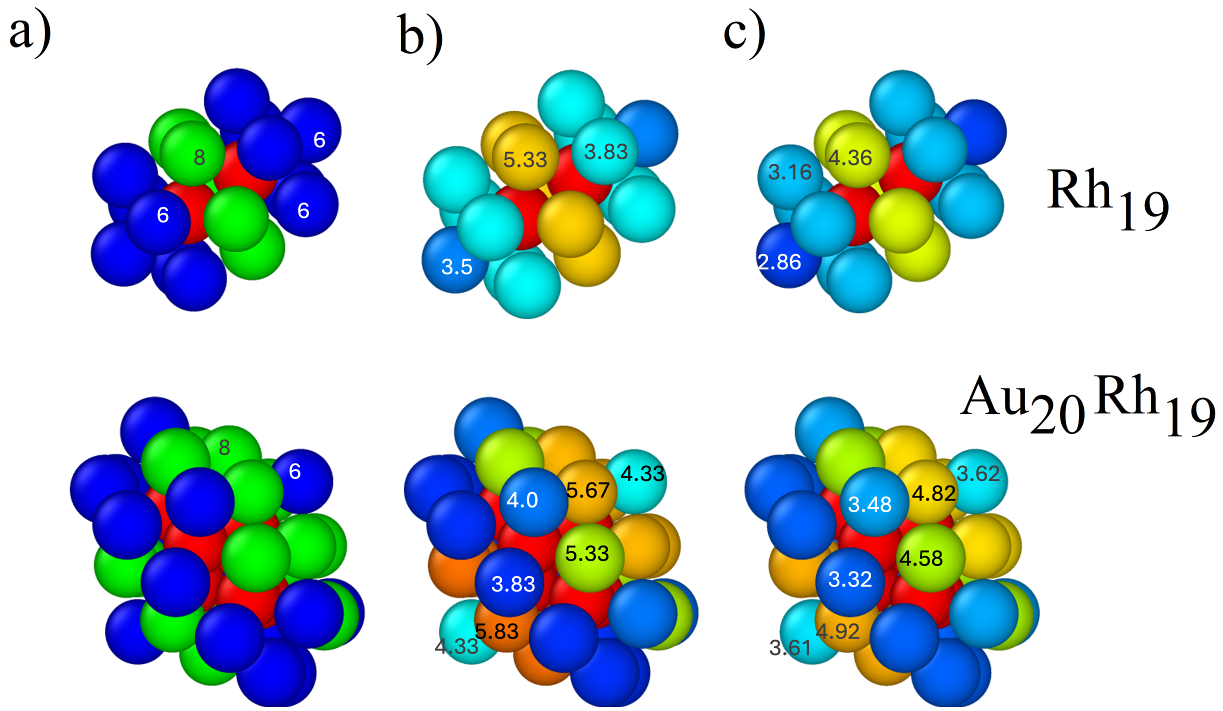

Fig. S8: Mapping for Rh_{19} $Au_{20}Rh_{19}$, $Au_{36}Rh_{19}$ and $Au_{52}Rh_{19}$. From left to right, figures show the nominal coordination, atop GCN considering a rigid cutoff of 3.08Å and sGCN.

Figure S9 shows the linear regression of the differences in adsorption energy, ΔE_{ad}, calculated between PBE+U and standard PBE values, as function of U values. In some cases, higher values of U have been considered to check the behavior of the systems. ΔE_{ad} shows a greater slope for hollow and bridge sites than the top. Furthermore, a change in the total magnetization has been observed for the Rh_{19}-CO_{t3} site. For PBE and U = 0.65 eV, the magnetization is 19 μ_B, while when U = 2.6 eV the system total magnetization is equal to 21 μ_B. This happens because the total magnetization depends on the difference between spin-up and spin-down electron densities. Since the U influences the energy of CO frontier orbitals, it also impacts the mixing between CO and metallic orbitals, thereby altering the shape of the two densities.

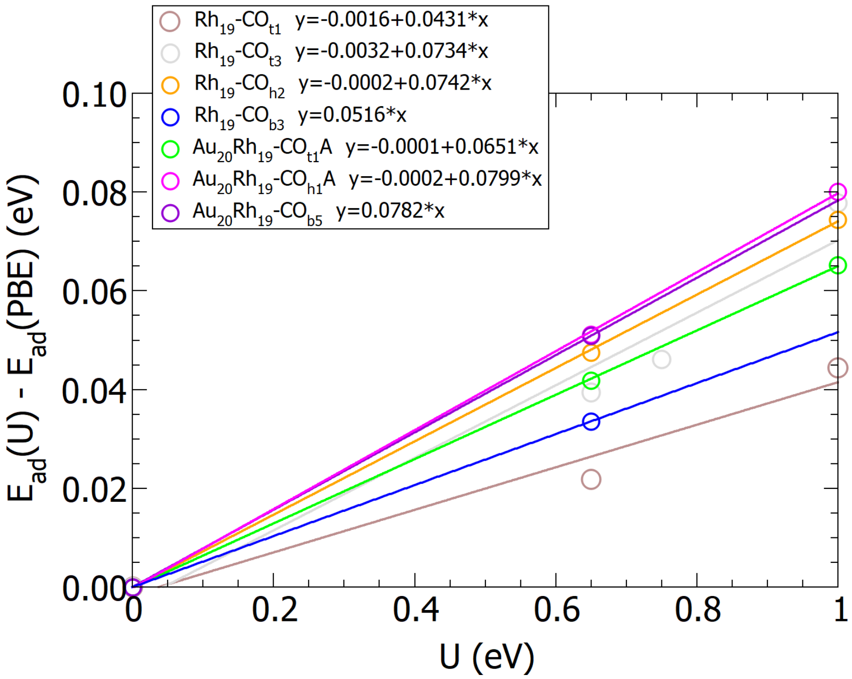

Fig. S9: Adsorption energy difference between values obtained at the PBE+Us and PBE levels. Data are fitted with lines.

Figure S10 shows that the DOS of the investigated nanosystems do not change sensibly with the accounting for the U. Figure S11 shows the pDOS magnification of CO p-states of the most stable top (t3) and hollow (h2) CO adsorption sites on Rh_{19}. As noted, the peaks just below the Fermi level, in the region of 4d orbitals, are greater when the CO is on hollow h2, compared to the top t3 adsorption site, and this holds for both, PBE and PBE+U2 levels of calculation. These results confirm that the 4d-2π* backdonation is greater when CO is on the hollow site, compared to the atop.

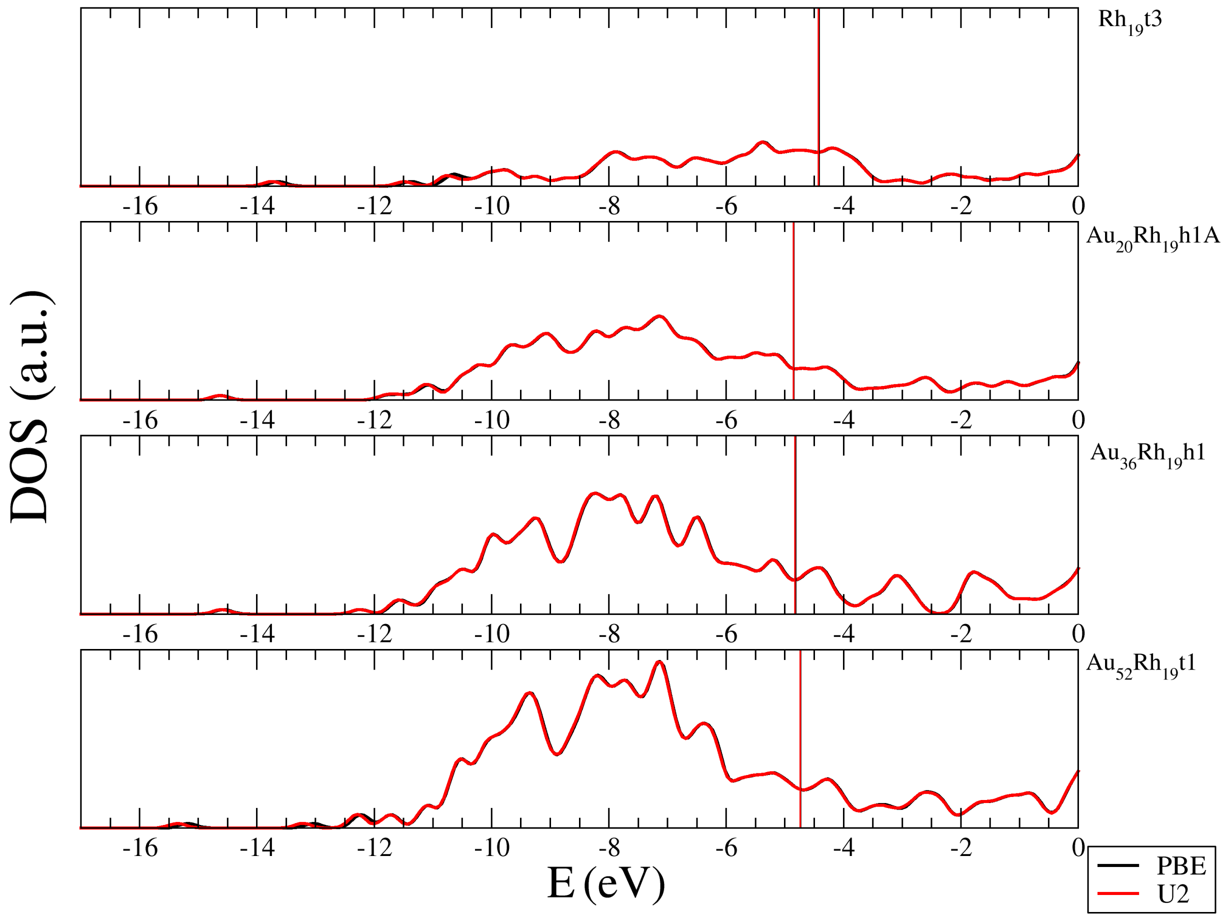

Fig. S10: DOS of the considered nanoalloys comparing PBE and PBE+U2. Zero energy represents the vacuum energy, and lines mark the Fermi energy.

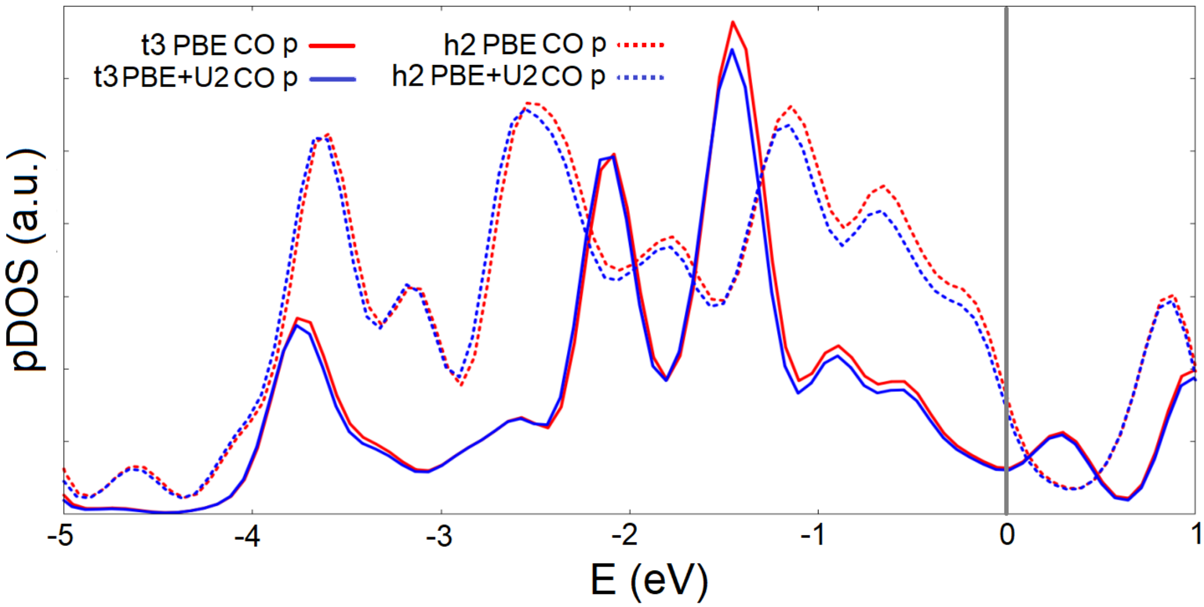

Fig. S11: pDOS of CO p-orbitals when the CO molecule is on t3 top and h2 hollow adsorption sites on the Rh_{19} NP at PBE and PBE+U2 levels of calculations. The Fermi level is at zero energy.

Figure S12 and S13 shows the Bader charge[15,16] redistribution ($\Delta q^i = q^i_{after} - q^i_{before}$) for the i atoms upon CO adsorption on the nanoparticles. The results refer to PBE+U1 for figure S12 and PBE+U2 for figure S13.

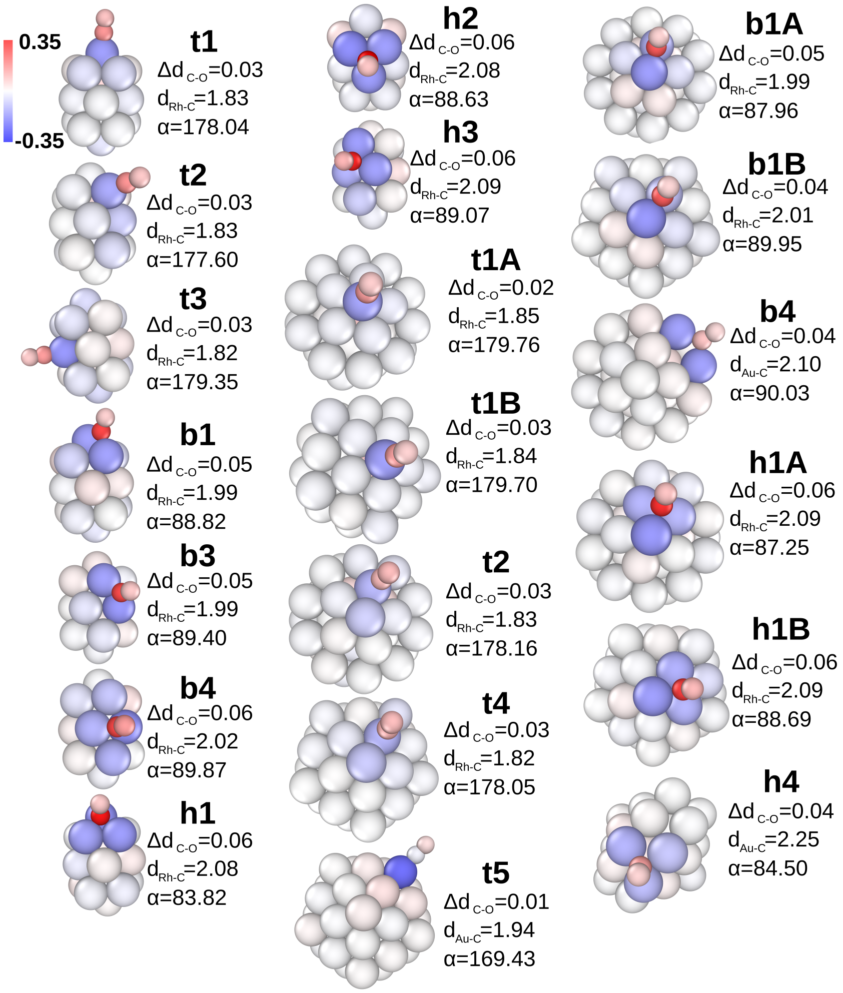

Fig. S12: PBE+U1 (U = 0.65 eV) charge redistribution and geometrical descriptors for CO on Rh_{19} and on $Au_{20}Rh_{19}$. Atoms are colored according to their charge redistribution (Δq^i), after and before the CO-addition. Blue atoms indicate electron depletion and red atoms indicate electron acquisition. Δd_{CO} and d_{Rh-C} are the CO bond length difference and the average Rh-CO distance after adsorption respectively. Both are given in Å . α indicates the angle in degrees between the CO axis and the plane containing the Rh-anchoring C.

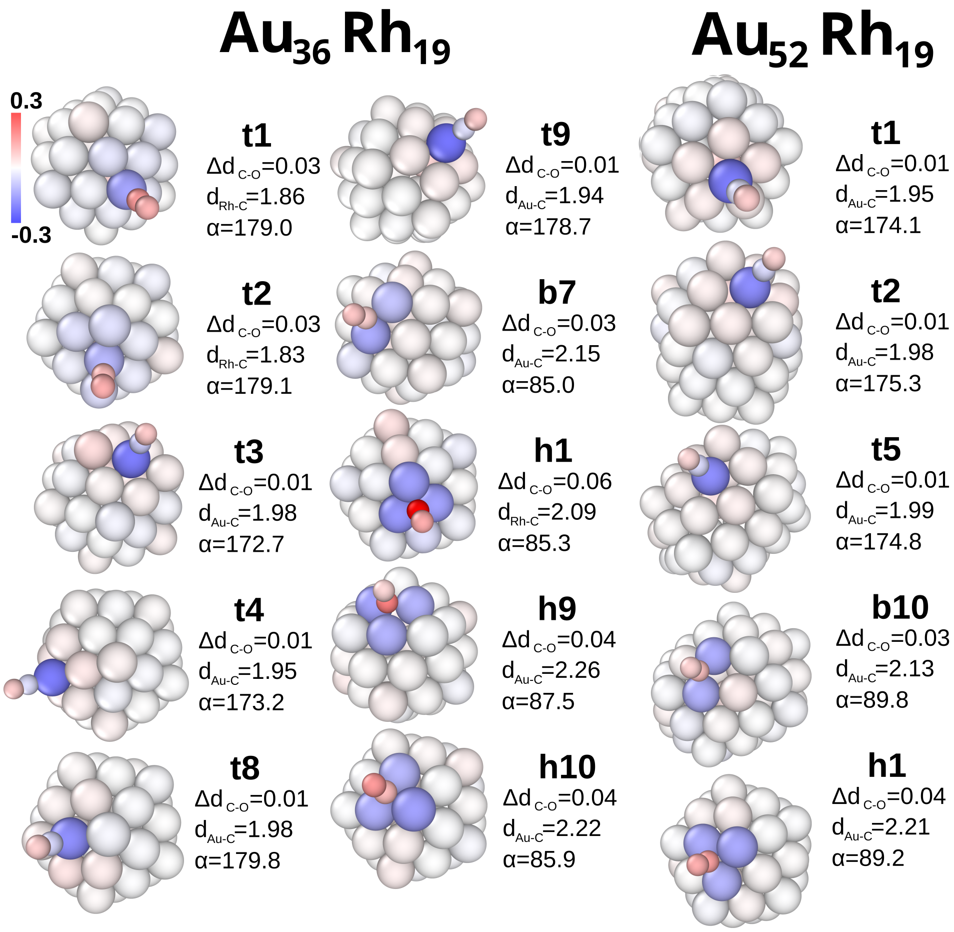

Fig. S13: PBE+U2 (U = 2.6 eV) charge redistribution and geometrical descriptors for CO on $Au_{36}Rh_{19}$ and on $Au_{52}Rh_{19}$. Atoms are colored according to their charge redistribution (Δq^i), after and before the CO-addition. Blue atoms indicate electron depletion and red atoms indicate electron acquisition. Δd_{CO} and $d_{Rh/Au-C}$ are the CO bond length difference and the average Rh/Au-CO distance after adsorption respectively. Both are given in Å . α indicates the angle in degrees between the CO axis and the plane containing the Rh-anchoring C.

Figure S14 shows the relation between adsorption energy and charge rearrangement on the molecule for Rh_{19}, while and Figure S15 for the $Au_{20}Rh_{19}$ nanostructure. As visible, there are no clear correlations. However, it is noticeable that smaller amounts of charge transferred to the molecule provide smaller adsorption energies, especially in the case of $Au_{20}Rh_{19}$.

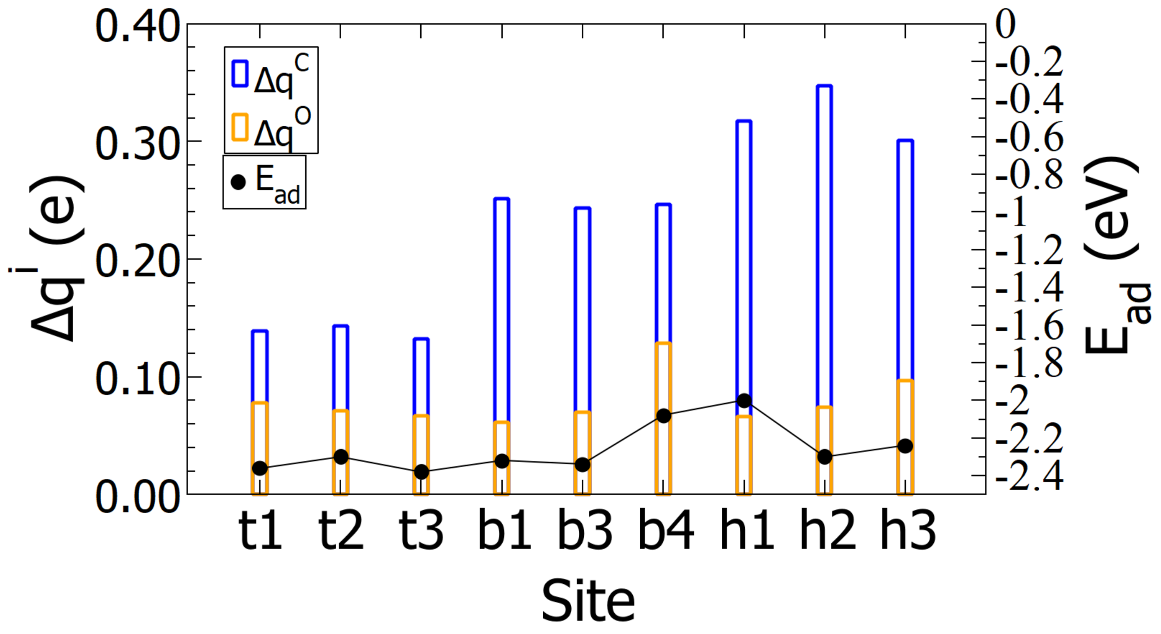

Fig. S14: Amount of charge acquired by C and O atoms upon molecular adsorption on Rh_{19} and corresponding adsorption energy. Results obtained at the PBE+U1 level.

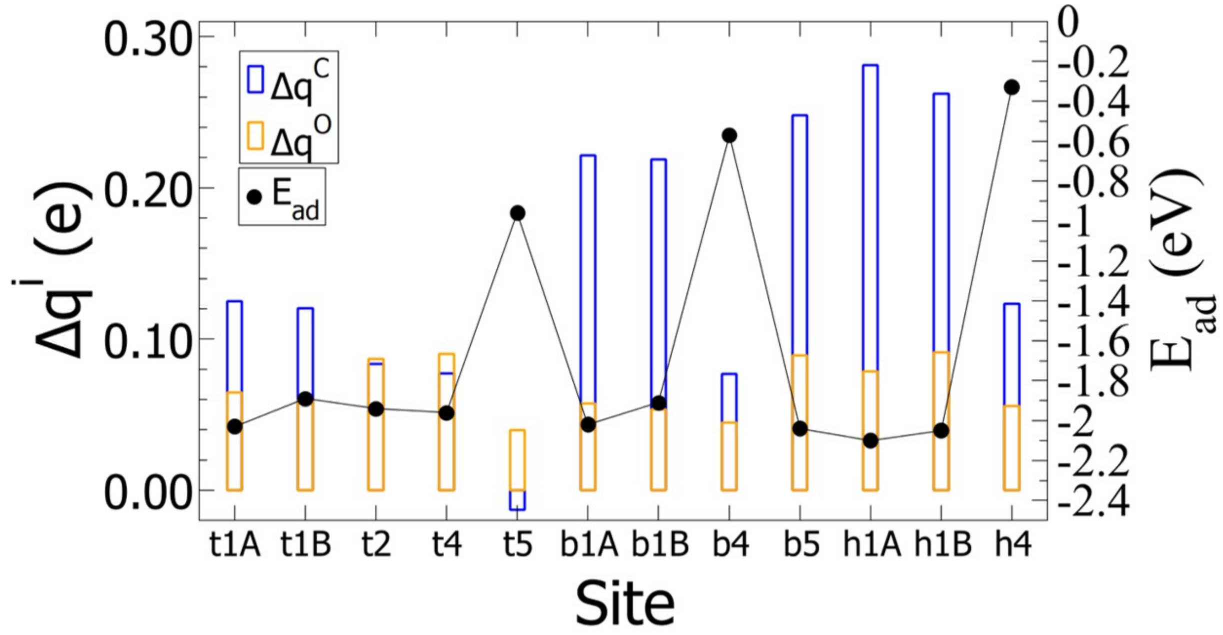

Fig. S15: Amount of charge acquired by C and O atoms upon molecular adsorption on $Au_{20}Rh_{19}$ and corresponding adsorption energy. Results obtained at the PBE+U1 level.

References

(1) Hammer, B.; Morikawa, Y.; Nørskov, J. K. CO Chemisorption at Metal Surfaces and Overlayers. *Phys. Rev. Lett.* **1996**, *76*, 2141–2144.

(2) Hammer, B.; N, J. *Impact of Surface Science on Catalysis*; Advances in Catalysis; Academic Press, 2000; Vol. 45; pp 71–129.

(3) Dudarev, S. L.; Botton, G. A.; Savrasov, S. Y.; Humphereys, C. J.; Sutton, A. P. Electron-energy-loss spectra and the structural stability of nickel oxide: An LSDA+U study. *Phys. Rev. B* **1998**, *57*, 1505.

(4) Cococcioni, M.; de Gironcoli, S. Linear response approach to the calculation of the effective interaction parameters in the LDA+U method. *Phys. Rev. B* **2005**, *71*, 035105.

(5) Belton, D. N.; Schmieg, S. J. Effect of Rh particle size on CO desorption from Rh/alumina model catalysts. *Surface Science* **1988**, *202*, 238–254.

(6) Wei, D.; Skelton, D.; Keva, S. Desorption and molecular interactions on surfaces: CO/Rh (110), CO/Rh (100) and CO/Rh (111). *Surface Science* **1997**, *381*, 49–64.

(7) Patra, A.; Peng, H.; Sun, J.; Perdew, J. P. Rethinking CO adsorption on transition-metal surfaces: Effect of density-driven self-interaction errors. *Phys. Rev. B* **2019**, *100*, 035442.

(8) Grimme, S.; Antony, J.; Ehrlich, S.; Krieg, H. A consistent and accurate ab initio parametrization of density functional dispersion correction (DFT-D) for the 94 elements H-Pu. *J. Chem. Phys.* **2010**, *132*, 154104.

(9) Araujo, R. B.; Rodrigues, G. L. S.; dos Santos, E. C.; Pettersson, L. G. M. Adsorption energies on transition metal surfaces: towards an accurate and balanced description. *Nature Communications* **2022**, *13*, 6853.

(10) Janthon, P.; Luo, S. A.; Kozlov, S. M.; Viñes, F.; Limtrakul, J.; Truhlar, D. G.; Illas, F. Bulk Properties of Transition Metals: A Challenge for the Design of Universal Density Functionals. *Journal of Chemical Theory and Computation* **2014**, *10*, 3832–3839.

(11) Illas, F.; López, N.; Ricart, J. M.; Clotet, A.; Conesa, J. C.; Fernández-García, M. Interaction of CO and NO with PdCu(111) Surfaces. *J. Phys. Chem. B* **1998**, *102*, 8017–8023.

(12) Linke, R.; Curulla, D.; Hopstaken, M. J. P.; Niemantsverdriet, J. W. CO/Rh(111): Vibrational frequency shifts and lateral interactions in adsorbate layers. *J. Chem. Phys.* **2001**, *115*, 8209–8216.

(13) Calle-Vallejo, F. The ABC of Generalized Coordination Numbers and Their Use as a Descriptor in Electrocatalysis. *Advanced Science* **2023**, *10*, 2207644.

(14) Calle-Vallejo, F.; Bandarenka, A. S. Enabling Generalized Coordination Numbers to Describe Strain Effects. *ChemSusChem* **2018**, *11*, 1824 – 1828.

(15) Sanville, E.; Kenny, S. D.; Smith, R.; Henkelman, G. Improved grid-based algorithm for Bader charge allocation. *Journal of Computational Chemistry* **2007**, *28*, 899–908.

(16) Tang, W.; Sanville, E.; Henkelman, G. A grid-based Bader analysis algorithm without lattice bias. *Journal of Physics: Condensed Matter* **2009**, *21*, 084204.
